# Adenovirus prevents dsRNA formation by promoting efficient splicing of viral RNA

**DOI:** 10.1093/nar/gkab896

**Published:** 2021-10-21

**Authors:** Alexander M Price, Robert T Steinbock, Chao Di, Katharina E Hayer, Yize Li, Christin Herrmann, Nicholas A Parenti, Jillian N Whelan, Susan R Weiss, Matthew D Weitzman

**Affiliations:** Division of Protective Immunity, Department of Pathology and Laboratory Medicine, The Children's Hospital of Philadelphia, Philadelphia, PA, USA; Division of Protective Immunity, Department of Pathology and Laboratory Medicine, The Children's Hospital of Philadelphia, Philadelphia, PA, USA; Cell & Molecular Biology Graduate Group, University of Pennsylvania, Philadelphia, PA, USA; Department of Biomedical and Health Informatics, The Children's Hospital of Philadelphia, Philadelphia, PA, USA; Department of Biomedical and Health Informatics, The Children's Hospital of Philadelphia, Philadelphia, PA, USA; Department of Microbiology, Perelman School of Medicine, University of Pennsylvania, Philadelphia, PA, USA; Division of Protective Immunity, Department of Pathology and Laboratory Medicine, The Children's Hospital of Philadelphia, Philadelphia, PA, USA; Cell & Molecular Biology Graduate Group, University of Pennsylvania, Philadelphia, PA, USA; Department of Microbiology, Perelman School of Medicine, University of Pennsylvania, Philadelphia, PA, USA; Department of Microbiology, Perelman School of Medicine, University of Pennsylvania, Philadelphia, PA, USA; Department of Microbiology, Perelman School of Medicine, University of Pennsylvania, Philadelphia, PA, USA; Division of Protective Immunity, Department of Pathology and Laboratory Medicine, The Children's Hospital of Philadelphia, Philadelphia, PA, USA; Department of Pathology and Laboratory Medicine, Perelman School of Medicine, University of Pennsylvania, Philadelphia, PA, USA

## Abstract

Eukaryotic cells recognize intracellular pathogens through pattern recognition receptors, including sensors of aberrant nucleic acid structures. Sensors of double-stranded RNA (dsRNA) are known to detect replication intermediates of RNA viruses. It has long been suggested that annealing of mRNA from symmetrical transcription of both top and bottom strands of DNA virus genomes can produce dsRNA during infection. Supporting this hypothesis, nearly all DNA viruses encode inhibitors of dsRNA-recognition pathways. However, direct evidence that DNA viruses produce dsRNA is lacking. Contrary to dogma, we show that the nuclear-replicating DNA virus adenovirus (AdV) does not produce detectable levels of dsRNA during infection. In contrast, abundant dsRNA is detected within the nucleus of cells infected with AdV mutants defective for viral RNA processing. In the presence of nuclear dsRNA, the cytoplasmic dsRNA sensor PKR is relocalized and activated within the nucleus. Accumulation of viral dsRNA occurs in the late phase of infection, when unspliced viral transcripts form intron/exon base pairs between top and bottom strand transcripts. We propose that DNA viruses actively limit dsRNA formation by promoting efficient splicing and mRNA processing, thus avoiding detection and restriction by host innate immune sensors of pathogenic nucleic acids.

## Graphical Abstract

**Graphical Abstract:**
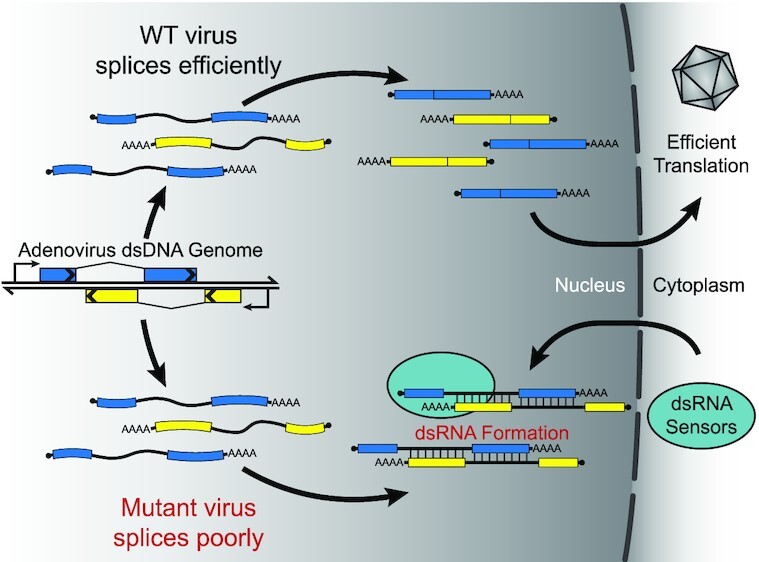
Inefficient splicing of overlapping viral transcripts can lead to intermolecular dsRNA formation. During wildtype virus infection the presence of the E1B55K/E4orf6 viral hijacked ubiquitin ligase leads to the ubiquitination of cellular RNA binding proteins (RBPs) RALY and hnRNPC, which precludes their binding to viral RNA. In the presence of RALY and hnRNPC viral transcripts are poorly spliced, leading to the formation of dsRNA between the exonic regions of viral RNAs to the intronic regions of transcripts derived from the opposing strand. After these dsRNA molecules form, a fraction of the cytoplasmic dsRNA-sensor PKR translocates into the nucleus, where it co-localizes with viral dsRNA and is activated by auto-phosphorylation.

## INTRODUCTION

To replicate productively, viruses must take over host intracellular processes while avoiding detection by the cellular innate immune system. Cells encode pattern recognition receptors (PRRs) that distinguish biomolecules indicative of an infected state (Pathogen associated molecular patterns, PAMPs) from otherwise normal metabolic processes (1). Ultimately, these PRRs promote an anti-microbial state by activation of type I or III interferon responses, or by directly shutting down processes necessary for pathogen and host survival (2). Many PRRs detect aberrantly structured or localized nucleic acids, such as cytoplasmic DNA, uncapped RNA, or highly structured RNA (3–5). In addition, there are multiple families of innate immune sensors that detect long stretches of complementary double-stranded RNA (dsRNA) (6). These include the RIG-I-like receptors (RLRs, RIG-I and MDA5) that activate interferon responses (2,7,8), the oligoadenylate synthases that produce 2–5A to activate ribonuclease L (OAS/RNaseL) and cleave viral and cellular single-stranded RNA (9–11), and the Protein Kinase RNA activated (PKR) protein that phosphorylates downstream translation initiation factors to block viral and cellular protein translation (12–14). Successful pathogens typically encode inhibitors of one or more of these pathways, resulting in a host/pathogen arms race (15–18).

Adenoviruses (AdV) are a family of nuclear-replicating, double-stranded linear DNA viruses that cause respiratory, ocular or enteric disease in humans (19). While symptoms are typically mild, certain strains of AdV can lead to severe acute respiratory distress or death, especially in immunocompromised individuals (20). Similar to other DNA viruses, AdV transcribes viral messenger RNAs from multiple promoters located on both the top and anti-sense bottom strands of DNA. Intermolecular annealing of these complementary strands of mRNA is thought to produce dsRNA that can trigger innate immune responses (21). This hypothesis is further supported by the fact that many DNA viruses encode inhibitors of dsRNA-responsive cellular pathways (9,12,22). However, many DNA viruses increase expression of endogenous retroelements which can also activate dsRNA sensors (23–26). Thus, it is unclear whether intermolecular viral dsRNA is formed during DNA virus infection. In fact, many DNA viruses actively prevent dsRNA formation since dsRNA is only detected during infection with viral mutants. Recently it was shown that cytoplasmic-replicating vaccinia virus produces dsRNA that can be blocked by viral decapping enzymes and host exonuclease activity (27,28). Nuclear-replicating herpes simplex virus can induce both nuclear and cytoplasmic dsRNA in the absence of US11 (a dsRNA-binding protein) or the virion host shutoff endonuclease VHS (29,30). Reactivation of the oncogenic herpesvirus KSHV is restricted by RIG-I (31), although the nature of host or viral RNAs bound by RIG-I in this context is controversial (32,33). Importantly, none of these reports have directly sequenced total dsRNA produced after infection to define their host or viral origin.

Compact viral genomes maximize coding capacity through extensive use of alternative promoters, splicing, and polyadenylation (19). Expression of these frequently overlapping viral transcripts relies on modulation of cellular factors such as RNA polymerase II and the spliceosome, exploiting multiple host RNA-binding proteins. We recently demonstrated that the efficiency of AdV splicing is regulated by a virus-directed ubiquitin ligase retargeted by viral proteins E4orf6 and E1B55K that ubiquitinates host RNA-binding proteins RALY and hnRNPC (34). In the absence of ubiquitination, viral late RNAs are bound by these cellular factors, are poorly spliced, and retained in the nucleus. Knockdown of RALY and hnRNPC restores efficient splicing of viral transcripts during ligase-deficient infection. Due to the genome organization of AdV, intron-containing unspliced transcripts are likely to form dsRNA. Prior biochemical methods detected similar amounts of dsRNA between infected and uninfected cells, and were not able to distinguish between structured (*cis*) and intermolecular (*trans*) dsRNA (35). Nevertheless, human AdVs are known to encode two highly structured, non-coding virus-associated RNAs (VA RNA) that block the activity of the dsRNA-sensor PKR (36,37). In addition, VA RNA has also been proposed to inhibit the activity of the dsRNA-sensor OAS1 *in vitro* (38).

In this study, we used two independent monoclonal antibodies to ascertain whether AdV produces dsRNA. We discovered that neither wildtype AdV nor a viral mutant deleted of VA RNA produce detectable levels of dsRNA during infection. However, we found that infection with AdV mutants lacking virus-directed ubiquitin ligase activity accumulated dsRNA in the nucleus that was dependent on late gene transcription. In the presence of dsRNA, the cytoplasmic sensor PKR relocalized to the nucleus and was activated even in the presence of inhibitory VA RNA. Pull-down and sequencing of dsRNA during infection confirmed a composition of poorly spliced viral RNA. Furthermore, we were able to induce the formation of viral dsRNA by modulating the splicing efficiency of wildtype virus, and also inhibit the formation of viral dsRNA with mutant viruses by removing host RNA binding proteins. These data highlight a novel mechanism by which viruses can escape innate immune recognition by modulating host factors to promote efficient viral RNA production.

## MATERIALS AND METHODS

### Cell culture

All cell lines were obtained from American Type Culture Collection (ATCC) and cultured at 37°C and 5% CO_2_. All cell lines tested negative for mycoplasma infection and were routinely tested afterwards using the LookOut Mycoplasma PCR Detection Kit (Sigma-Aldrich). A549 cells (ATCC CCL-185) were maintained in Ham's F-12K medium (Gibco, 21127-022) supplemented with 10% (v/v) FBS (VWR, 89510-186) and 1% (v/v) Pen/Strep (100 U/ml of penicillin, 100 μg/ml of streptomycin, Gibco, 15140-122). HeLa cells (ATCC CCL-2), U2OS cells (ATCC HTB-96), Vero cells (CCL-81), IMR90 cells (CCL-186) and HEK293 cells (ATCC CRL-1573) were grown in DMEM (Corning, 10–013-CV) supplemented with 10% (v/v) FBS and 1% (v/v) Pen/Strep. HBEC-KT3 cells (ATCC CRL-4051) were grown in Airway Epithelial Cell Basal Medium (ATCC PCS-300-030) supplemented with Bronchial Epithelial Cell Growth Kit (ATCC PCS-300–040) and 1% (v/v) Pen/Strep.

### Viral infection

Adenovirus serotype 5 (Ad5) was originally purchased from ATCC. Ad5 ΔE1B55K mutant dl110 (39) was previously described and obtained from G. Ketner. Ad5 ΔE4 mutant dl1004 (40) and dl366 (41) were previously described and obtained from G. Ketner and D. Ornelles. Ad5 ΔE4 mutants with re-addition of E4orf3 (dl366 + orf3) (42) or E4orf4 (dl366 + orf4) (42) were obtained from D. Ornelles. Ad5 ΔE4orf1-3 (dl1006) (40) was previously described and obtained from G. Ketner. Ad5 ΔE4orf6 (dl355*) (42) was previously described and obtained from D. Ornelles. Ad5 ΔVA I/II (dl720) (43) was previously described and obtained from C. Sullivan. ΔE4 viruses were expanded and titered on complementing W162 vero cells (44). ΔVA virus were expanded and titered on PKR KO A549 cells. All other viruses were expanded and titered on HEK293 cells. All viruses were purified using two sequential rounds of ultracentrifugation in cesium chloride gradients and stored in 40% (v/v) glycerol at −20°C (short term) or −80°C (long term). Viral stock titer was determined by plaque assay, and all subsequent infections were performed at a multiplicity of infection (MOI) of 10 PFU/cell unless stated otherwise. Cells were infected at 80–90% confluent monolayers by incubation with diluted virus in a minimal volume of low serum (2%) media for two hours. After infection viral inoculum was removed by vacuum and full serum growth media was replaced for the duration of the experiment.

### Antibodies and inhibitors

The following antibodies were used for cellular proteins: Total PKR clone Y117 (Abcam ab32506, WB: 1:1000, IF 1:500), phosphorylated PKR threonine 446 clone E120 (Abcam ab32036, WB: 1:1000), phosphorylated PKR threonine 451 (Abcam ab81303, IF 1:500), total eIF2α (Cell Signaling 9722S, WB 1:500), phosphorylated eIF2α serine 51 (Cell Signaling 9721S, WB 1:500), RALY (Bethyl Laboratories A302–070A, WB 1:1000), hnRNPC (Santa Cruz Biotechnology sc-32308, WB 1:1000), RAD50 (GeneTex GTX70228, WB 1:1000), CUL5 (Bethyl Laboratories A302-173A, WB 1:200), USP7 (Bethyl Laboratories A300-033A, IF 1:500), Histone H3 (Abcam ab1791, WB 1:10 000) and GAPDH (GeneTex GTX100118, WB 1:30 000). Antibodies against dsRNA include J2 (Scicons 10010200, IF 1:500), mouse 9D5 (EMD Millipore 3361, IF 1:2), and rabbit 9D5 (Absolute Antibody Ab00458-23.0, IF 1:1000). Primary antibodies against viral proteins were obtained from: rabbit polyclonal against adenovirus Hexon, Penton, and Fiber (Gift from J. Wilson, WB 1:10 000), mouse anti-DBP (Gift from A. Levine, Clone: B6-8, WB 1:1000, IF 1:400), polyclonal rabbit anti-DBP (Gift from A. Levine, IF: 1:40 000), rabbit anti-protein VII (Gift from H. Wodrich, IF 1:200), and mouse anti-E1B55K (Gift from A. Levine, Clone: 58K2A6, WB 1:500).

NEDDylation inhibitor MLN4924 was purchased from Sigma-Aldrich (505477), resuspended in dimethyl sulfoxide (DMSO), and used at a final concentration of 3 μM added 12 hpi. RNA polymerase inhibitor actinomycin D was purchased from Cayman Chemical (11421), resuspended in DMSO, and used at a final concentration of 5 μM added 24 hpi. 5,6-Dichloro-1-β-d-ribofuranosyl-1H-benzimidazole (DRB) was purchased from Cayman Chemical (10010302), resuspended in DMSO, and used at a final concentration of 20 μM added 24 hpi. RNA pol III inhibitor ML60218 was purchased from EMD Millipore (557403), resuspended in DMSO, and used at a final concentration of 30 μM added 24 hpi.

### siRNA and morpholino transfections

The following siRNA pools were obtained from Dharmacon: non-targeting control (D-001206–13-05), RALY (M-012392–00-0005) and hnRNPC (M-011869-01-0005). All siRNA transfections were performed using the standard protocol for Lipofectamine RNAiMAX (Invitrogen). Antisense morpholinos were designed using the splice modifying pre-mRNA target designer and purchased from Gene Tools. Final sequences can be found in Supplementary Table S1. 15 μM of late transcript targeting Morpholinos (TPL, 5 μM each) or non-targeting control were delivered to cells two hours post viral infection using 6 μM PEG Endo-Porter (Gene Tools OT-EP-PEG-1) per manufacturer's directions. Non-targeting control Morpholino was designed against GFP and purchased from Gene Tools (PCO-GFPControl-100). Poly(I:C) was provided pre-complexed with transfection reagent (Invivogen tlrl-piclv), and was reconstituted fresh with molecular grade water before addition to cells at a concentration of 500 ng/ml

### PKR Knockout

PKR KO A549s were constructed as previously published (45). In brief, guide RNAs targeting PKR (Forward primer: CACCGTAATACATACCGTCAGAAGC; Reverse primer: AAACGCTTCTGACGGTATGTATTAC) were selected from previously published sgRNA database (46). Primers were annealed and cloned into pLenti-CRISPR (Addgene) using published methods (47). Pseudo lentiviruses were packaged in HEK293 cells using pLenti-CRIPSR (with sgRNA), psPAX2 and pCMV-VSV-G. Infectious supernatants were transferred to A549 cells and selected using 2 μg/ml of puromycin for 3 days. Puromycin-resistant cells were cloned by limited dilution.

### Immunoblotting and subcellular fractionation

For western immunoblotting protein samples were prepared by directly lysing cells in lithium dodecyl sulfate (LDS) loading buffer (NuPage) supplemented with 1% beta-mercaptoethanol (BME) and boiled at 95°C for 10 min. Subcellular fractionation into cytoplasmic and nuclear compartments was performed using the REAP method before lysis in LDS (48). In brief, cells were washed and pelleted before being triturated in ice-cold 0.1% NP40 in PBS. A portion of this lysate was retained as the whole cell fraction, and then pulse-centrifuged on a tabletop microcentrifuge to separate the cytoplasmic fraction into the supernatant and the ‘nuclear’ fraction into the pellet. Equal volumes of protein lysate were separated by SDS-PAGE in MOPS buffer (Invitrogen) before being transferred onto a nitrocellulose membrane (Millipore) at 35 V for 90 min in 20% methanol solution. Membranes were stained with Ponceau to confirm equal loading and blocked in 5% (w/v) non-fat milk in TBST supplemented with 0.05% w/v sodium azide. Membranes were incubated with primary antibodies in milk overnight, washed for three times in TBST, incubated with HRP-conjugated secondary (Jackson Laboratories) for 1 h and washed an additional three times in TBST. Proteins were visualized with Pierce ECL or Pico Western Blotting Substrate (Thermo Scientific) and detected using a Syngene G-Box. Images were processed and assembled in Adobe Photoshop and Illustrator CC 2021.

### Indirect immunofluorescence assays and analysis

Cells were grown on glass coverslips in 24-well plates, mock-infected or infected with virus for the appropriate time, washed twice with PBS and then fixed in 4% (w/v) Paraformaldehyde for 15 min. Cells were permeabilized with 0.5% (v/v) Triton-X in PBS for 10 min, and blocked in 3% (w/v) BSA in PBS (+ 0.05% w/v sodium azide) for one hour. Primary antibody dilutions were added to coverslips in 3% (w/v) BSA in PBS (+ 0.05% w/v sodium azide) for 1 h, washed with 3% BSA in PBS three times, followed by secondary antibodies and 4,6-diamidino-2-phenylindole (DAPI) for one hour. Secondary antibodies were used at 1:500 dilution and conjugated to Alexa-Fluor 488 (Invitrogen A-11001 or A-11008), or Alexa-Fluor 568 (Invitrogen A-11004 or A-11011) in anti-mouse or anti-rabbit. Coverslips were mounted onto glass slides using ProLong Gold Antifade Reagent (Cell Signaling Technologies). Immunofluorescence was visualized using a Zeiss LSM 710 Confocal microscope (Cell and Developmental Microscopy Core at UPenn) and ZEN 2011 software. Images were processed in FIJI (v1.53c) and assembled in Adobe Photoshop and Illustrator CC 2021. Quantification of nuclear dsRNA intensity was performed in FIJI. In brief, individual nuclei were masked by thresholding the DAPI channel after subtracting background and running a gaussian blur. Nuclei masks were then applied to the dsRNA channel where the ‘Measure’ function was iteratively called for each cell. Measure produced values for nuclei size, minimum intensity, maximum intensity, and average intensity which provided the mean nuclear fluorescence intensity (MNFI).

### RNA isolation and qRT-PCR

Total RNA was isolated from cells by either TRIzol extraction (Thermo Fisher) or RNeasy Micro kit (Qiagen), following manufacturer protocols. Cytoplasmic RNA was extracted using RLN Buffer (50 mM Tris–HCl pH 8.0, 140 mM NaCl, 1.5 mM MgCl_2_, 0.5% (v/v) Nonidet P-40) followed by the manufacturers protocol from Qiagen. RNA was treated with RNase-free DNase I (Qiagen), either on-column or after ethanol precipitation. RNA was converted to complementary DNA (cDNA) using 1 μg of input RNA in the High Capacity RNA-to-cDNA kit (Thermo Fisher). Quantitative PCR was performed using the standard protocol for SYBR Green reagents (Thermo Fisher) in a QuantStudio 7 Flex Real-Time PCR System (Applied Biosystems). All primers were used at 10 μM and sequences can be found in Supplementary Table S1. All values were normalized by the ΔΔCt method by normalizing first to internal controls such as GAPDH and HPRT1.

### dsRNA immunoprecipitation and dsRNA RIP-Seq

Anti-dsRNA enrichment was adapted from previously published methods (49,50). Total RNA was harvested from WT Ad5 or ΔE4-infected cells 48 hpi by TRIzol extraction. RNA was diluted to 500 ng/μl in NET2 Buffer (50 mM Tris–HCl pH 7.6, 150 mM NaCl, 3 mM MgCl_2_) and divided into immunoprecipitated fractions (IP, 50 μg RNA) or 1% input RNA (0.5 μg RNA). IP samples were treated with 2 units of diluted single-strand specific RNase I (Thermo AM2294, 100 U/μl) and 2 μl TURBO DNase (Thermo AM2238) at 37°C for 10 min before the addition of 10 μl SuperAse RNase inhibitor (Thermo AM2696) and placement on ice. 5 μg J2 anti-dsRNA antibody (Scicons) was added to IP fraction to a final volume of 200 μl and incubated overnight at 4°C with end over end rotation. Protein G Dynabeads (Thermo 10004D) were washed twice with NET2 before being blocked with 1 mg/ml BSA and 10 μg/ml linear acrylamide (Thermo AM9520) for thirty minutes. Blocked beads were added to J2/RNA mixture and allowed to continue rotating for an additional two hours. RNA-bound beads were subsequently washed using ice-cold NET2 buffer thrice for five minutes each before RNA was extracted using TRIzol LS (Thermo 10296010). Aqueous-phase TRIzol was subsequently extracted using RNA Clean and Concentrator (Zymo Research R1015) and eluted into 11 μL elution buffer. Input and dsRNA-enriched fractions were converted into cDNA and analyzed by qRT-PCR as a fraction of input RNA, or immediately converted into Illumina sequencing libraries as detailed below.

### RNA sequencing and analysis

Input or dsRNA-enriched RNA from above was converted into Illumina sequencing libraries using the NEBNext Ultra II Directional RNA library kit (NEB E7760S) following manufacturers instructions with the following modifications. Ribosomal RNA was depleted using NEBNext rRNA depletion kit v2 (NEB E7405S). Input RNA was chemically fractionated for 15 min, while dsRNA IP RNA was fractionated for 7 min as directed for partially degraded RNA due to prior RNase I digestion. IDT xGen UDI-UMI Adapters 1–8 (Integrated DNA Technologies 10006913) were ligated to resulting cDNA after second-strand synthesis and end-prepping. All library purification steps used NEBNext RNA Sample Purification Beads (Beckman Coulter Agencourt RNAClean XP) or SPRISelect (Beckman Coulter B23317). After initial library quantification via qPCR, final libraries were amplified using xGen Library Amplification Primer Mix (Integrated DNA Technologies 1076775) and NEBNext Ultra II Q5 master mix for 4 cycles (input libraries) or 10 cycles (IP libraries). Libraries were quantified using Thermo Qubit fluorometric quantification, Agilent Bioanalyzer DNA Chip and qPCR quantification relative to a standard curve. Eight total libraries were pooled in equimolar ratios and loaded onto an Illumina NextSeq 500 High Output flow cell (Illumina FC-404-2005) and ran for 150 cycles in single end mode.

Using Picard Tools (version 2.23.9) modules ExtractIlluminaBarcodes, IlluminaBasecallsToSam and SamToFastq, Illumina basecall (BCL) files were demultiplexed to bam, to keep track of the unique molecular identifiers (UMIs) for each read, and to fastq files for further processing. Raw reads were subsequently mapped simultaneously to the human (hg38) and Ad5 (AC_000008.1) genomes using STAR (version 2.7.6a). The alignment files were merged (Picard Tools module MergeBamAlignment) with the bam files from the first step to retrieve UMI information. Uniquely mapped reads were de-duplicated using umi_tools (version 1.0.1). STAR was run with the parameter –quantMode GeneCounts to quantify genes as annotated in gencode v35. Splice junctions were extracted and annotated from the above results using RegTools (version 0.5.2). The read counts were normalized to the total number of unique mapped de-duplicated reads per library. For global viral splicing analysis, a particular junction was only considered as real if there were at least two unique reads spanning the junction.

## RESULTS

### Wildtype adenovirus does not produce detectable dsRNA

**Figure 1.**
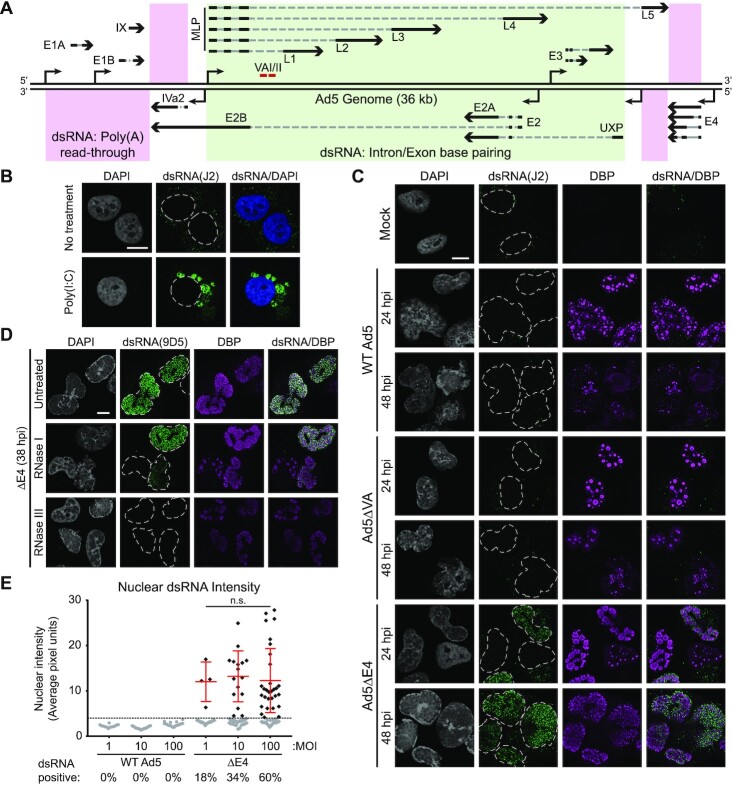
Detection of dsRNA only during infection with mutant viruses. **(A**) Schematic of the AdV transcriptome showing major promoters, directionality, and strand of major transcriptional units. Exons are shown in black, introns in dashed grey lines, and polyadenylation sites shown by arrowheads. Potential regions that can form dsRNA in *trans* by poly(A) read-through (pink) or intron/exon base-pairing (green) are highlighted. (**B**) dsRNA can be detected by the monoclonal antibody J2. After transfection of A549s with 500 ng/mL poly inosine:cytosine, dsRNA can be detected accumulated in the cytoplasm. (**C**) dsRNA was stained with J2 (green) and co-stained with viral replication center (VRC) marker DBP (magenta). Nuclei were stained with DAPI, and nuclear periphery were outlined by a dashed white line. A549 cells were infected with WT Ad5, or Ad5 mutants lacking VA RNA (ΔVA, dl720) or the entire E4 region (ΔE4, dl1004) at MOI of 10 for 24 or 48 h post infection (hpi). (**D**) A549 cells were infected with ΔE4 for 38 h before fixation and permeabilization. Cells were treated for 30 min with single-stranded RNase (RNase I) or double-stranded RNase (RNase III) at 37C before being stained against dsRNA (9D5, green) or VRC marker DBP (magenta). (**E**) A549 cells were infected with WT or ΔE4 virus for 48 h at the listed multiplicities of infection (MOI) and stained for dsRNA by IFA as in (C). Nuclear dsRNA was quantified as Mean Nuclear Fluorescence Intensity (MNFI) and every cell displayed as a dot. The dsRNA positive threshold was defined as 4 standard deviations over the MNFI of dsRNA in uninfected cells and displayed as a dashed line. Cells below the threshold were colored gray, while cells positive for nuclear dsRNA were colored black and the mean and standard deviation of these positive cells is denoted by red error bars. The percentage of total cells expressing dsRNA during infection with each virus is shown below their name on the x-axis. Statistical significance was derived from an unpaired two tailed Mann–Whitney *t*-test, where n.s. denotes not significant. For all IFA, scale bars (white line) display 10 μm.

Adenovirus serotype 5 (Ad5) contains five early transcriptional units, two intermediate transcriptional units, and two late transcriptional units derived from 10 promoters, with extensive processing through alternative splicing and polyadenylation (51). While these transcripts are produced from both top and bottom strands of the DNA genome and share anti-sense complementarity, the majority of the long-lived exonic RNA species do not overlap with a complementary exon from the opposite strand. Instead, annealed dsRNA would be more likely to form with intron/exon base pairing of unprocessed transcripts from the core of the transcriptome, or from polyadenylation read-through products at the genomic termini (Figure 1A). To assay for dsRNA production during infection we performed indirect immunofluorescence assays (IFA) on A549 human adenocarcinoma lung cells using the J2 anti-dsRNA monoclonal antibody (52). In uninfected A549s, this antibody detects dim cytoplasmic dots which are known to co-localize with mitochondria (53), and reveals cytoplasmic aggregates after transfection with the dsRNA mimic poly inosine:cytosine (pI:C, Figure 1B). Upon infection of A549 cells with wildtype (WT) Ad5 we did not detect any dsRNA staining at either early (24 h) or late (48 h) times post-infection (Figure 1C). Likewise, we saw no evidence of dsRNA when cells were infected with an Ad5 mutant lacking VA RNA (ΔVA, dl720), a known and potent inhibitor of the dsRNA sensor PKR (43) (Figure 1C). We then used mutant viruses to explore the hypothesis that large amounts of viral dsRNA accumulate only when poor RNA processing allows for intron/exon base pairing. We infected A549s with a mutant AdV lacking the viral products produced from the E4 region (ΔE4, dl1004), which is known to yield poorly processed and highly unstable viral RNAs (40,41,54). In this scenario, we now detected robust production of dsRNA that was localized within the nucleus (Figure 1C). These results suggest that accumulation of significant dsRNA is prevented during WT Ad5 infection through the function of viral E4 proteins.

We next sought to confirm that lack of dsRNA detection was not due to antibody cross-reactivity, or an inaccessible epitope of the J2 monoclonal antibody. To this end, we employed an orthogonal anti-dsRNA antibody, 9D5, which has been previously reported to be more sensitive than the J2 antibody for dsRNA (55,56). Upon infecting A549 cells with WT Ad5 we were still unable to detect dsRNA (Supplementary Figure S1A). However, 9D5 once again revealed a similar intranuclear pattern of dsRNA staining upon infection with the ΔE4 virus. Co-staining confirmed that J2 and 9D5 detected the same intranuclear sites of dsRNA accumulation (Supplementary Figure S1B). To rule out cross-reactivity to other nucleic acid structures, we confirmed that these antibodies are indeed detecting dsRNA, as fluorescence staining was retained after pre-treating fixed cells with the single-strand specific nuclease RNase I but abolished by treatment with the double-strand specific nuclease RNase III (Figure 1D, Supplementary Figure S1C).

We then asked what role changing viral load might have upon dsRNA formation during infection. While our standard multiplicity of infection (MOI) of 10 plaque-forming units per cell results in nearly 100% of cells being infected, we tested both low viral load (MOI 1, less than every cell infected) and very high viral load (MOI 100, saturating infection). We did not detect dsRNA accumulation at any dose of WT Ad5, but both low- and high-dose ΔE4-infection resulted in cells with readily detectable nuclear dsRNA (Figure 1E). Interestingly, while the brightness of detected dsRNA was not significantly different between low- or high-dose ΔE4-infection, the number of cells positive for dsRNA increased with increasing viral load. Although infections of mutant viruses at high MOI can frequently complement some of their defects, we hypothesize that in this case more transcriptionally-competent viral genomes leads to increased production of dsRNA in the absence of E4 viral proteins.

Finally, we further examined WT and ΔE4 infections in a diverse array of cell types to rule out that this production of dsRNA was an artifact of the particular cancer cell line used. Cell lines tested included cervical cancer HeLa, osteosarcoma U2OS, human embryonic kidney HEK293, African green monkey kidney epithelial Vero, normal lung fibroblast IMR90, and immortalized human bronchial epithelial cells HBEC-KT3 (Supplementary Figure S1D). In all instances infection with WT Ad5 yielded no cells with evidence of dsRNA production, whereas ΔE4-infection always produced a fraction of cells positive for dsRNA. This implies that the lack of dsRNA detection during WT Ad5 infection is not cell-type or species-specific.

### Cells infected with mutant virus produce dsRNA upon entry into the late phase of infection

**Figure 2.**
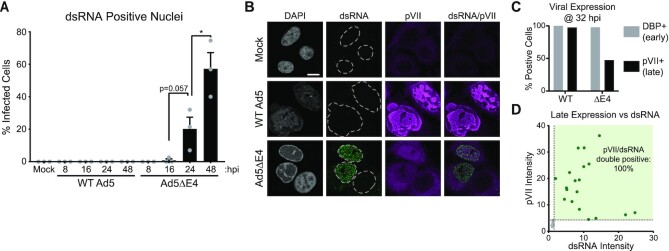
dsRNA formation correlates with entry into the late phase of infection. (**A**) The percentage of infected cells expressing nuclear dsRNA from three independent biological replicates are plotted as a function of hours post-infection. Positivity was defined as 4 standard deviations over the MNFI of cells in the uninfected condition. Bars depict mean, and error bars denote standard deviation. Total number of cells analyzed ranged from 114 to 203, with an average of 176 cells per condition. Significance was determined by unpaired two-tailed *t*-test, where * denotes *P*-value <0.05. (**B**) A549 cells were infected with WT or ΔE4 Ad5 for 32 h and co-stained for dsRNA (green) and viral late protein pVII (magenta). Scale bar (white line) displays 10 μm. (**C**) Quantification of cells from the experiment shown in (B) that were positive for viral early antigen (DBP+, gray) or viral late antigen (pVII+, black). (**D**) Scatterplot of ΔE4-infected cells from (B) highlighting both dsRNA and pVII expression. While only ∼50% of infected cells display dsRNA, 100% of late protein expressing cells display dsRNA. Positive thresholds for both dsRNA and pVII were defined as 4 standard deviations above the MNFI of uninfected cells.

In any given cell type or time post-infection we observed that only a subset of cells infected with ΔE4 virus showed evidence of dsRNA production. Over a time course of infection, the fraction of dsRNA-positive cells increased steadily, with essentially no detection of dsRNA at 8 or 16 h post infection (hpi), to ∼20% at 24 h post infection, and >50% positive at 48 hpi (Figure 2A). To ascertain why only a fraction of ΔE4-infected cells became dsRNA positive, we co-stained with additional viral antigens, including the late viral protein pVII (Figure 2B). At high MOI, essentially 100% of WT Ad5-infected cells were positive for both early (DBP) and late (pVII) antigens at 32 hpi (Figure 2C). In contrast, the ΔE4 mutant is defective for the late stage of infection, and we observed that only ∼50% of infected cells became positive for the late marker pVII. By comparing co-stains for pVII and dsRNA in ΔE4 infected cells, we demonstrated that 100% of cells with detectable dsRNA were also positive for pVII, whereas infected cells that had not reached the late stage of infection were negative for dsRNA (Figure 2D). These data support the hypothesis that dsRNA forms after the major late promoter (MLP) is activated during mutant virus infection.

### Viral ubiquitin ligase activity prevents dsRNA formation

**Figure 3.**
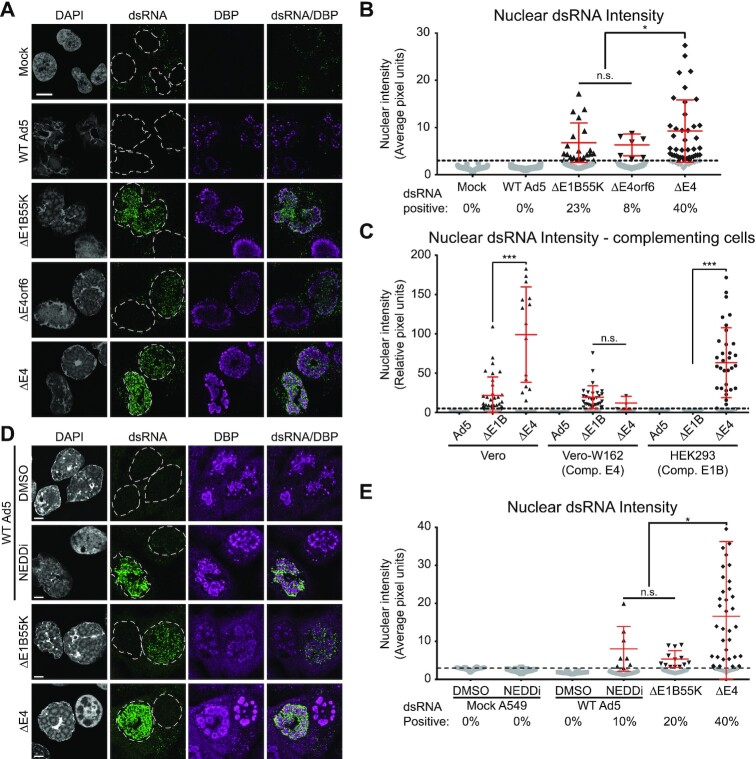
Viral ubiquitin ligase activity prevents dsRNA formation. (**A**) dsRNA was stained with J2 (green) and co-stained against VRCs marked by DBP (magenta). A549 cells were mock-infected or infected with WT Ad5, or Ad5 mutants lacking E1B55K (ΔE1B55K, dl110), E4orf6 (ΔE4orf6, dl355*), or the entire E4 region (ΔE4, dl1004) at MOI of 10 for 38 h. (**B**) Nuclear dsRNA from (A) was quantified as MNFI and every cell displayed as a dot. The dsRNA positive threshold was defined as 4 standard deviations over the MNFI of dsRNA in uninfected cells and displayed as a dashed line. Cells below the threshold were colored grey, while cells positive for nuclear dsRNA were colored black and the mean and standard deviation of these positive cells is denoted by red error bars. The percentage of total cells expressing dsRNA during infection with each virus is shown below their name on the x-axis. (**C**) Cells complementing various aspects of the AdV ubiquitin ligase were infected at an MOI of 10 for 32 h and dsRNA was detected as in (A) and quantified as in (B). Vero cells do not complement AdV genes, and dsRNA was therefore detected during infection with both ΔE1B55K (ΔE1B) and ΔE4 mutant viruses. Vero-W162 contains the full AdV E4 region under control of the endogenous E4 promoter, and complements ΔE4 but not ΔE1B55K virus infection. HEK293 cells contain the AdV E1A and E1B gene products and complement ΔE1B55K virus infection. (**D**) IFA against dsRNA and DBP was performed as in (A), however the WT Ad5 was split into two conditions treated with DMSO vehicle control or 3 μM of the NEDDylation inhibitor MLN-4924 (NEDDi) added at 12 hpi. Infected A549 cells were fixed at 30 hpi. (**E**) Nuclear dsRNA from (D) quantified as in (B). For all IFA, scale bars (white line) display 10 μm. Statistical significance was derived from an unpaired two tailed Mann–Whitney *t*-test, where n.s. denotes not significant, * denotes *P*< 0.05, *** denotes *P*< 0.001.

We next sought to ascertain which viral proteins within the multifunctional E4 region were necessary to prevent dsRNA formation. The most well-characterized functions of individual E4 proteins include mislocalization of antiviral restriction factors by E4orf3 (40,41), and degradation of antiviral proteins by the retargeting of a host ubiquitin ligase through the combined actions of E4orf6 and E1B55K (57–59). The E4orf4 product can also modulate diverse cellular processes, including RNA transcription and splicing, through effects on cellular signaling pathways (60). Two independently derived deletions of the E4 region (dl1004 and dl366) produced dsRNA after infection of A549 cells (Supplementary Figure S2A). Infection with a virus mutant deleted of only E4orfs 1–3 (dl1006) did not produce any dsRNA, and adding back just E4orf3 or E4orf4 into the complete ΔE4 was not sufficient to limit dsRNA production. However, mutants with single deletions of virus-directed ubiquitin ligase components E4orf6 (dl355*) or the targeting subunit E1B55K (dl110 or dl1520) were sufficient to generate dsRNA (Figure 3A). Interestingly, compared to the full ΔE4 deletion these two individual deletions led to a smaller fraction of dsRNA-positive cells, as well as less intense dsRNA fluorescence (Figure 3B). Together these data suggest that other functions of the E4 proteins, while not sufficient on their own, may synergize with the loss of the AdV ubiquitin ligase to allow AdV-induced dsRNA formation.

Both E4orf6 and E1B55K are known to have roles in viral infection independent of each other and their ubiquitin ligase activity (61). To address whether these features could be provided *in trans*, we employed specific complementing cell lines. The W162 line is an engineered Vero cell that expresses the full E4 region under control of the endogenous E4 promoter (44). While infection of this cell line complemented E4 function to reduce dsRNA accumulation from that observed during ΔE4 virus infection, it was not able to limit dsRNA production resulting from deletion of the genetically distinct E1B55K component of the ubiquitin ligase (Figure 3C). Conversely, HEK293 cells, which are immortalized by integration of the E1A and E1B regions of AdV (62), can limit dsRNA production during ΔE1B55K virus infection, but not during ΔE4 infection. Thus, both E4orf6 and E1B55K components of the AdV ubiquitin ligase must be present in the cell to prevent dsRNA formation during virus infection.

As an orthogonal method to confirm the necessity of ubiquitin ligase function, we chemically inhibited the host CUL5 protein necessary for viral ubiquitin ligase activity by blocking the NEDD8 post-translational modification of Cullins with the inhibitor MLN4924 (63). While inhibiting NEDDylation likely impacts all Cullin RING ligases (64), this is beneficial to our study of the AdV ubiquitin ligase since the virus can use multiple Cullin proteins (65). Inhibition of NEDDylation was confirmed by loss of the slow migrating modified CUL5 band by immunoblot analysis, the prevention of RAD50 degradation, and relative loss of viral late protein production (Supplementary Figure S2B). Strikingly, we saw dsRNA production for the first time in an otherwise WT infection when ubiquitin ligase activity was inhibited (Figure 3D). The percentage of cells and intensity of dsRNA produced after NEDDylation inhibition were similar to that seen in ΔE1B55K infection (Figure 3E). Together, these data implicate the ubiquitin ligase activity of AdV in actively preventing dsRNA formation after infection.

### PKR is activated by nuclear dsRNA in the presence of viral PKR inhibitor

**Figure 4.**
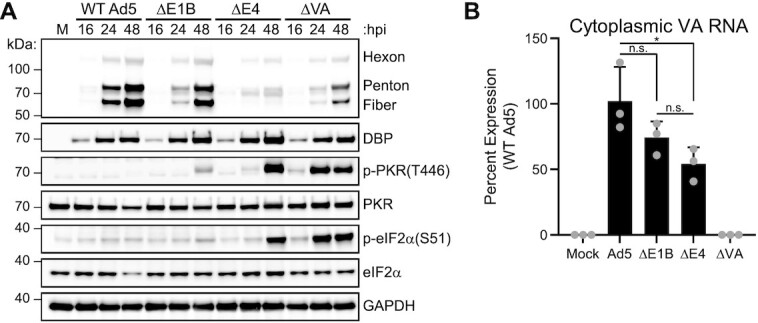
PKR can be activated by nuclear dsRNA even in the presence of VA RNA. (**A**) Time-course immunoblot following infection of A549 cells with WT Ad5, or Ad5 mutants lacking E1B55K (ΔE1B, dl110), the E4 region (ΔE4, dl1004), or both VA RNAs (ΔVA, dl720) at MOI of 10. Activation of the PKR pathway is shown by auto-phosphorylation of PKR at threonine 446 and downstream phosphorylation of eIF2α at serine 51. (**B**) A549 cells were infected for 32 h, and cytoplasmic RNA was harvested and converted into cDNA. Quantitative PCR for VA RNA I was performed, normalized to GAPDH levels, and set with WT Ad5 at 100% expression. Bars depict mean, and error bars show standard deviation of three biological replicates (grey dots). Statistics were performed using unpaired two-tailed *t*-tests where *P* > 0.05 was not significant (n.s.) and *P*< 0.05 is denoted as *.

It has been previously reported that infection with Ad5 mutants lacking the ubiquitin ligase can result in activation of the dsRNA sensor PKR, however dsRNA production was not shown in that study (66). To further investigate this finding, we performed immunoblot analysis over a time-course of infection with WT Ad5, and compared to mutants lacking E1B55K, the entire E4 region, or both VA RNAs (Figure 4A). PKR activation is evident by auto-phosphorylation of threonine residue 446, and downstream signaling was demonstrated by phosphorylation of eIF2α serine residue 51. For both ΔE1B and ΔE4 mutant viruses, activation of PKR was observed late during infection, coincident with our detection of dsRNA production (Figures 4A and 2A). Phosphorylation of PKR and eIF2α was observed much earlier for ΔVA virus infection (16–24 h) as previously reported (67), even in the absence of detectable dsRNA (Figure 1C). We next sought to rule out the possibility that our mutant viruses which produce dsRNA have fewer or mislocalized VA RNAs. We confirmed that infections of both ΔE1B- and ΔE4 viruses expressed similar amounts of VA RNA compared to WT virus, and that these RNAs were properly exported to the cytoplasm (Figure 4B). It has been previously reported that PKR knockdown is sufficient to rescue late protein synthesis during ΔVA infection (68). We confirmed this finding in Cas9-mediated PKR knockout (PKR KO) A549 cells where the late protein defect of ΔVA infection was partially rescued (Supplementary Figure S3A). PKR KO was not sufficient to rescue the late protein defect of ΔE4-infected cells, highlighting that in this system dsRNA production is upstream of PKR activation.

### Cytoplasmic PKR is relocalized to the nucleus after dsRNA formation

**Figure 5.**
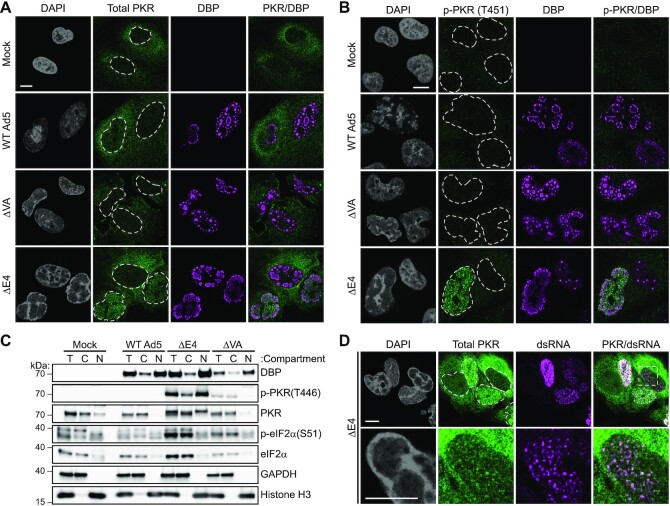
PKR is relocalized to the nucleus after dsRNA formation during virus infection. (**A**) A549 cells were infected with WT Ad5, or Ad5 mutants lacking E1B55K (ΔE1B, dl110), lacking both VA RNAs (ΔVA, dl720), or lacking the E4 region (ΔE4, dl1004) at MOI of 10 for 38 h. IFA was performed and cells were stained for total PKR (green) and VRC marker DBP (magenta). Nuclei were stained with DAPI, and nuclear periphery are outlined by a dashed white line. (**B**) A549 cells were infected as in (A) and stained for activated PKR (phosphorylated threonine 451, green) and VRC marker DBP (magenta). (**C**) A549 cells were infected for 32 h and subcellular fractionation performed to analyze cytoplasmic (C) or nuclear (N) compartments. A portion of input cells were reserved for total (T) cellular lysis. Fractionation quality was confirmed by cytoplasmic marker GAPDH and nuclear marker histone H3. (**D**) A549 cells were infected with ΔE4 virus for 38 h and stained for total PKR (green) and 9D5 anti-dsRNA antibody (magenta). Nuclei were stained with DAPI, and nuclear periphery were outlined by a dashed white line. Zoomed region (White box) displays small puncta of dsRNA without PKR, or dsRNA co-localizing with puncta of nuclear PKR. For all IFA, scale bars (white line) display 10 μm.

Considering that PKR is typically considered to be a cytoplasmic sensor of dsRNA, we were curious as to how this sensor was activated by the nuclear dsRNA detected during mutant AdV infection. IFA showed the expected predominantly cytoplasmic localization of total PKR in uninfected A549 cells. Infection with WT and ΔVA viruses did not impact PKR localization. In contrast, a fraction of the total PKR signal relocalized to the nucleus in a subset of ΔE4-infected cells (Figure 5A). The percentage of ΔE4-infected cells with nuclear PKR (∼36%) was reminiscent of the amount of cells showing evidence of dsRNA at this timepoint. In contrast, cells infected with either WT- or ΔVA viruses showed no substantial increase in nuclear PKR compared to uninfected cells (Supplementary Figure S3B). Activation of PKR, as read out by phosphorylation of threonine 451, was only seen in the nuclear compartment of ΔE4-infected cells (Figure 5B). This relocalization was confirmed by subcellular fractionation and immunoblot analysis (Figure 5C). Interestingly, phosphorylated PKR was detected exclusively in the cytoplasm of ΔVA virus-infected cells and appeared too faint or too diffuse to localize by IFA. In ΔE4 virus-infected cells, the intranuclear fraction of total PKR and all activated PKR specifically localized to sites of dsRNA (Figure 5D and Supplementary Figure S3C). The appearance of dsRNA in ΔE4 virus-infected cells appears to precede PKR nuclear relocalization, since the highlighted cell appears to show few puncta of dsRNA staining without concomitant nuclear PKR staining (Figure 5D). These data demonstrate that nuclear dsRNA produced during ΔE4 virus infection is sensed by an endogenous cellular sensor, and induces its relocalization to the nucleus. Furthermore, our data highlight important differences in PKR activation, localization, and downstream effects between AdV mutants that produce dsRNA (ΔE4) versus ΔVA virus infection that does not produce detectable dsRNA.

### Nuclear dsRNA contains viral unspliced transcripts

**Figure 6.**
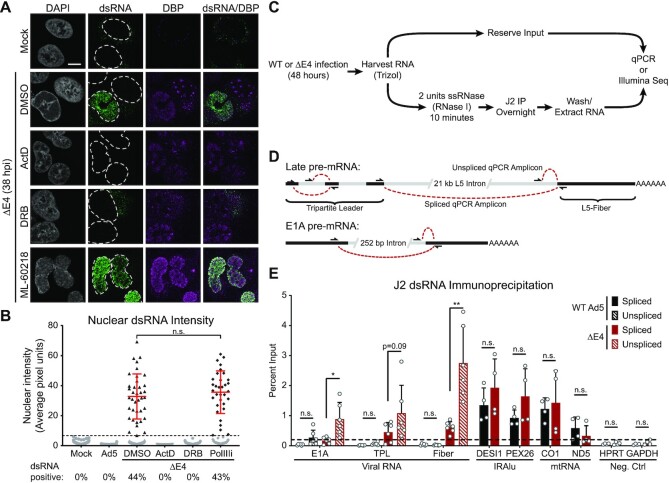
Nuclear dsRNA includes unspliced viral transcripts. (**A**) A549 cells were infected for 38 h and stained with 9D5 anti-dsRNA antibody (green) and VRC marker DBP (magenta). Nuclei were stained with DAPI, and nuclear periphery outlined by a dashed white line. Scale bar (white line) denotes 10 μm. ΔE4-infected cells were treated with DMSO vehicle, or treated with 5 μM polymerase inhibitor Actinomycin D (ActD), 20 μM RNA Pol II elongation inhibitor DRB, or 30 μM RNA Pol III inhibitor ML-60218 from 24 hpi until fixation. (**B**) Nuclear dsRNA from (A) was quantified as MNFI and every cell displayed as a dot. The dsRNA positive threshold was defined as 4 standard deviations over the MNFI of dsRNA in uninfected cells and displayed as a dashed line. Cells below the threshold were colored grey, while cells positive for nuclear dsRNA were colored black and the mean and standard deviation of these positive cells is denoted by red error bars. The percentage of total cells expressing dsRNA during infection with each virus is shown below the x-axis. Mann–Whitney *t*-test showed no significant change (n.s.) between either condition with positive dsRNA. (**C**) Workflow depicted for dsRNA immunoprecipitation using J2 antibody. (**D**) Diagram depicting qRT-PCR strategy for assaying both spliced and unspliced regions of viral transcripts for E1A, tripartite leader (TPL), and TPL-spliced L5-Fiber (Fiber). Exons are shown as black lines while introns are shown as grey lines. (**E**) Total RNA was harvested from WT Ad5 (black bars) or ΔE4-infected (red bars) A549s at 48 hpi and subjected to dsRNA immunoprecipitation with J2 antibody. RNA species are analyzed by qRT-PCR and normalized as a percentage of the specific transcript detected within the matching input RNA. Viral RNAs were analyzed as both spliced (solid bars) and unspliced (dashed bars), and compared to known positive control cellular dsRNA such as mRNA with inverted repeat Alu elements (IRAlu) or mitochondrial RNA (mtRNA). Positive threshold (dashed line) was set as 2-fold greater than the average of two negative controls HPRT1 and GAPDH. Bars depict mean of six independent biological replicates (viral transcripts) or four independent biological replicates (host transcripts) and error bars show standard deviation. Unpaired two-tailed *t*-tests were used to determine significance between conditions, where n.s. denotes *P*-value >0.05, * denotes *P*-value <0.05, and ** *P*< 0.01.

While nuclear dsRNA produced during AdV infection is localized around viral replication centers reminiscent of sites of RNA Pol II-dependent viral RNA synthesis (69), antibody staining does not prove the dsRNA we detect is viral in origin. One alternative source of dsRNA is the RNA Pol III-dependent expression of nuclear repetitive elements, which are upregulated by many DNA viruses including AdV (24). To investigate the source of the dsRNA, we treated ΔE4-infected A549 cells with various RNA polymerase inhibitors. Treatment with high concentration actinomycin D (ActD) broadly inhibits RNA polymerase activity and resulted in a total loss of dsRNA production when added at 24 hpi, implying that dsRNA production requires active transcription (Figure 6A). The CDK9 inhibitor 5,6-Dichloro-1-beta-D-ribofuranosylbenzimidazole (DRB) specifically blocks RNA Pol II elongation. DRB treatment of ΔE4-infected cells entirely blocked nuclear dsRNA production, while sparing the cytoplasmic dsRNA puncta indicative of mitochondrial RNA transcription (Figure 6A). The RNA Pol III specific inhibitor ML-60218 did not block the intensity nor percentage of cells expressing dsRNA (Figure 6A, B). These data suggest that dsRNA requires active RNA Pol II transcription from viral genomes and is not composed of RNA Pol III-dependent endogenous retroelement transcripts.

To determine whether viral transcripts are enriched in dsRNA, we used the J2 antibody to immunoprecipitate dsRNA from total RNA derived from WT Ad5 or ΔE4 virus-infected A549 cells (49,50) (Figure 6C). Primers designed to distinguish between spliced and unspliced forms were used for qRT-PCR analysis of three viral RNA species (70): E1A (viral early gene), tripartite leader (TPL, 3 exon sequence located 5′ of all viral late transcripts), and Fiber (most distal late gene) (Figure 6D). The RNA enriched by J2 immunoprecipitation was plotted as a percentage of input RNA, and compared to cellular positive controls for inverted repeat Alu-element containing mRNAs (71) (IRAlu) and mitochondrial RNAs (53) (mtRNA). All positive controls and all ΔE4-derived viral RNAs were enriched over the threshold defined by negative control housekeeping genes HPRT1 and GAPDH (Figure 6E). Furthermore, unspliced viral RNAs were more abundant than the respective spliced forms within the dsRNA fraction from ΔE4-infected cells. There was no significant difference in the enrichment of cellular positive controls between WT Ad5 and ΔE4-infection. These data indicate that dsRNA produced during ΔE4 AdV infection is most likely generated by complementary intron/exon base pairing of viral transcripts derived from opposing genome strands.

### Viral dsRNA is depleted for splice junctions across the transcriptome

**Figure 7.**
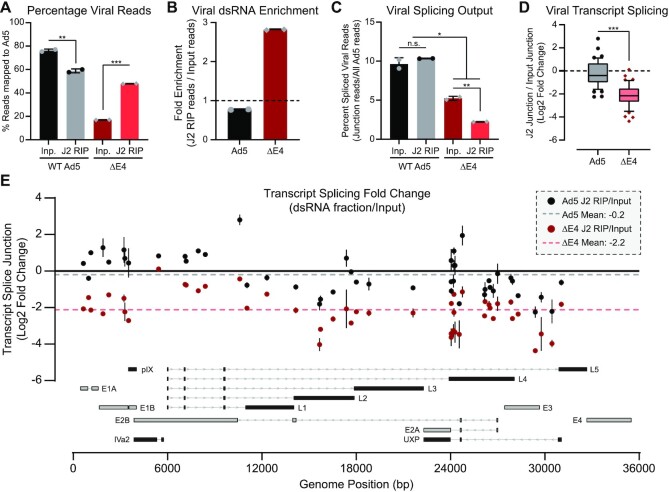
The RNA from ΔE4 virus infection is enriched for dsRNA and depleted of splice junctions across the transcriptome. (**A**) J2-immunoprecipitated RNA and matched input RNA were collected from two biological replicates each of Ad5- or ΔE4-infected A549s 48 hpi and Illumina sequencing was performed (diagrammed in Fig 6C). Percentage of all unique, de-duplicated reads mapping to the Ad5 genome within each input (Inp., black or red) or J2 dsRNA-enriched RNA immunoprecipitation (J2 RIP, gray or salmon) per sequencing library are shown, and data points depict two sequenced biological replicates with bars representing mean and error bars representing standard deviation. (**B**) The enrichment of dsRNA within viral reads was calculated by determining the fold change between the percentage of viral RNA discovered in the J2 RIP library and the percentage of viral RNA in the matched input library from the same biological replicate. Bars show the mean of the data with error bars depicting standard deviation. No enrichment for dsRNA would lead to a ratio of 1, which is depicted as a dotted black line. (**C**) Spliced viral reads were defined as an RNA read mapping to the viral genome and containing a reproducible splice junction (junction read being detected more than once per library and in all eight libraries), and were normalized as a percentage of the total number of mappable viral reads in each library. Data depict two sequenced biological replicates each and bars and error bars represent mean and standard deviation. (**D**) Forty seven specific splice junctions were chosen as being indicative of a single highly expressed viral transcript. Junctional abundance was normalized to the total mapped reads per library and log_2_ fold change was calculated between each junction within the J2 RIP and matching Input from the same biological replicate. Box and whisker plots show median, interquartile range, and 10th–90th percentiles. **(E)** The log2 fold change of each of the 47 splice junctions from (D) is displayed as a colored dot (WT Ad5, black; ΔE4, red) with a vertical black line showing the standard deviation between two biological replicate libraries. Splice junctions are distributed across the x-axis corresponding to the genomic locations of whichever splice donor or splice acceptor would yield the greatest spread of data. Mean fold change was calculated independently for WT Ad5 (dashed grey line) or ΔE4 (dashed salmon line). Major viral transcriptional units are shown to scale along the x-axis and consist of early transcripts (grey) and late transcripts (black). Major introns are shown as light gray lines with arrows indicating the direction of transcription. Mann–Whitney *t*-test was used to determine significance between conditions. For all statistical tests, * denotes *P*-value < 0.05, ** *P*< 0.01, *** *P*< 0.001.

To expand our analysis of virus-generated dsRNA, we performed J2 dsRNA immunoprecipitation (RIP) coupled to Illumina sequencing. By 48 hpi with WT Ad5, the majority of total RNA (ribosomal RNA-depleted) was viral in origin (75%), while viral RNA only constituted 17% of all RNA during ΔE4 virus-infection (Figure 7A). Relative to input RNA, however, viral RNA was enriched in J2-precipitated libraries during ΔE4 virus infection, while being relatively depleted in the analogous libraries from WT infections (Figure 7B). To analyze viral splicing, we utilized the ability of strand-specific sequencing to unambiguously map reads containing reproducible splice junctions, and compared these to the total amount of viral reads detected in each library. Approximately 10% of all viral RNA reads contained at least one splice junction in both the input and dsRNA-enriched libraries from WT Ad5 infection (Figure 7C). This percentage of total splicing was less for ΔE4-derived viral RNAs in the input libraries (∼5%) and substantially lower in the dsRNA-enriched libraries from ΔE4 virus-infection (∼2.5%) (Figure 7C). While our qRT-PCR strategy above was only able to unambiguously detect pre-mRNA from three viral RNAs, sequencing allowed for the selection of 47 specific viral splice junctions that were indicative of a specific high-abundance viral mRNA. To compare whether the splicing of specific transcripts was altered in dsRNA-enriched fractions, the fold-change of these transcript-surrogate junctions from the J2 RIP were normalized to the junction abundance in each sample's respective input. While all viral mRNA derived from WT Ad5 infection had essentially the same splicing index as compared to input RNA (mean log_2_ fold change –0.2), the dsRNA enriched from ΔE4 virus-infection contained significantly fewer spliced transcripts than total ΔE4 RNA (mean log_2_ fold change –2.12) (Figure 7D). This relative depletion of splicing was evenly spread across the viral transcriptome without specific hotspots (Figure 7E). Taken together, these data showcase that ΔE4 virus-infection is significantly enriched for viral dsRNA production compared to WT infection. Furthermore, the viral transcripts found within dsRNA are especially poorly spliced, even when compared to input RNA, suggesting that inefficient splicing enables the formation of complementary intron/exon base-pairing.

### Efficient viral RNA splicing prevents dsRNA formation

**Figure 8.**
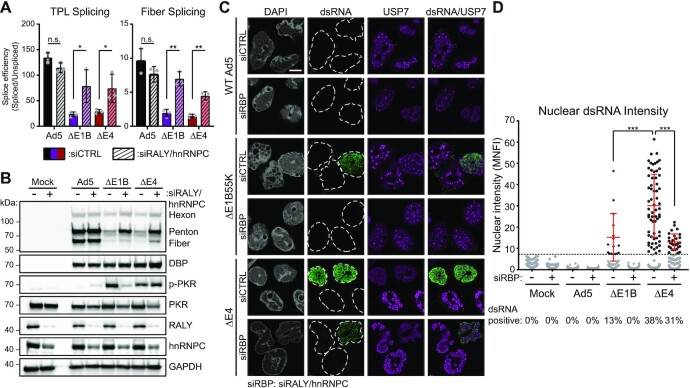
Efficient viral RNA splicing prevents dsRNA formation. (**A**) HeLa cells were transfected with control siRNA (solid bars) or siRNA targeting both RALY and hnRNPC (dashed bars) for 24 h before being infected with WT Ad5 (Ad5, black), or Ad5 mutants lacking E1B55K (ΔE1B, purple) or the E4 region (ΔE4, red). At 32 hpi total RNA was harvested and qRT-PCR was performed. Spliced and unspliced viral transcripts were detected using the method shown in 6D and the ratio of these RNA species depicted as splicing efficiency. Data are shown for three independent biological replicates, bars depict mean, and error bars depict standard deviation. Significance was analyzed by unpaired two-tailed *t*-test. (**B**) Immunoblot with exactly matching conditions to those shown in (A). (**C**) HeLa cells were transfected with control siRNA (siCTRL) or siRNA targeting both RALY and hnRNPC (siRBP) for 24 h before being infected with WT Ad5, ΔE1B or ΔE4. Cells were fixed at 32 hpi and stained with 9D5 anti-dsRNA antibody (green) and cellular protein that marks VRCs (USP7, magenta). Nuclei were stained with DAPI, and nuclear periphery are outlined by a dashed white line. Scale bar (white line) denotes 10 μm. (**D**) Nuclear dsRNA from (C) was quantified as MNFI and every cell displayed as a dot. The dsRNA positive threshold was defined as 4 standard deviations over the MNFI of dsRNA in uninfected cells and displayed as a dashed line. Cells below the threshold were colored grey, while cells positive for nuclear dsRNA were colored black, and the mean and standard deviation of these positive cells is denoted by red error bars. The percentage of total cells expressing dsRNA during infection with each virus is shown below their name on the x-axis. Significance was analyzed by unpaired two-tailed Mann–Whitney *t*-test. For all experiments, significance was shown as *P*-value >0.5 (not-significant, n.s.), * *P*< 0.05, ** *P*< 0.01 and *** *P*< 0.001.

AdV-directed ubiquitin ligase activity is necessary for efficient processing of viral mRNA (34). We therefore assessed whether rescue of viral RNA processing would be sufficient to block dsRNA production during infection with mutant viruses lacking components of the ubiquitin ligase. We previously showed that the AdV ligase ubiquitinated cellular RNA binding proteins hnRNPC and its homolog RALY (34). In the absence of ubiquitination these RBPs bind viral transcripts and prevent their efficient processing, but this phenotype can be reversed by knockdown of both factors. We confirmed that siRNA-mediated knockdown of RALY and hnRNPC was sufficient to rescue the splicing defect for upstream late transcript TPL leader and downstream Fiber splicing events in cells infected with ΔE4 as well as ΔE1B55K mutants (Figure 8A). This knockdown-induced rescue of viral splicing efficiency was sufficient to partially rescue viral late protein production in both mutant virus infections. This rescue was accompanied by a reduction in the amount of activated PKR during ΔE1B55K virus infection (Figure 8B). We assayed for production of dsRNA using IFA after RALY/hnRNPC knockdown and discovered that we could no longer detect dsRNA in ΔE1B55K virus-infected cells (Figure 8C). While ΔE4 virus-infected cells still produced dsRNA, the signal was much less intense and the percent of cells positive for dsRNA decreased, potentially explaining the partial rescue of late protein production observed by immunoblot analysis in the presence of activated PKR (Figure 8D). These data suggest that it is specifically the ability to promote efficient viral RNA splicing that limits the production of dsRNA.

**Figure 9.**
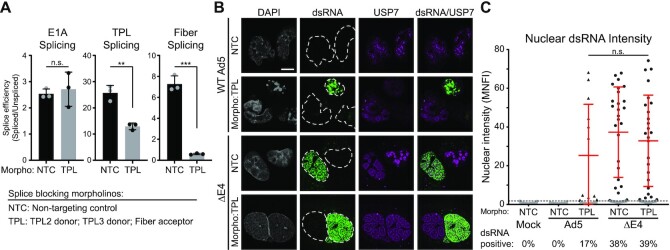
Blocking viral RNA splicing is sufficient to induce dsRNA formation. (**A**) A549 cells were infected with WT Ad5 and two hours later transfected with 15 μM non-targeting Morpholinos (NTC) or a cocktail of Morpholinos designed to block the TPL2 splice donor, TPL3 splice donor and Fiber splice acceptor (TPL, 5 μM each morpholino). At 24 hpi total RNA was harvested and viral RNA splice efficiency was calculated as in 8A. Data are shown for three independent biological replicates, bars depict mean, and error bars depict standard deviation. Significance was analyzed by unpaired two-tailed *t*-test. (**B**) A549s were infected with WT Ad5 or ΔE4, transfected with the Morpholinos from (A) at 2 hpi, and fixed for IFA at 24 hpi. Cells were stained with 9D5 anti-dsRNA antibody (green) and cellular protein that marks VRCs (USP7, magenta). Nuclei were stained with DAPI, and nuclear periphery are outlined by a dashed white line. Scale bar (white line) denotes 10 μm. (**C**) Nuclear dsRNA from (B) was quantified as MNFI and every cell displayed as a dot. The dsRNA positive threshold was defined as 4 standard deviations over the MNFI of dsRNA in uninfected cells and displayed as a dashed line. Cells below the threshold were colored gray, while cells positive for nuclear dsRNA were colored black and the mean and standard deviation of these positive cells is denoted by red error bars. The percentage of total cells expressing dsRNA during infection with each virus is shown below their name on the x-axis. Significance was analyzed by unpaired two-tailed Mann–Whitney *t*-test. For all experiments, significance was shown as *P*-value >0.5 (not-significant, n.s.), ** *P*< 0.01 and *** *P*< 0.001.

We next asked whether it was possible to impair viral RNA processing during WT Ad5 virus infection, and whether this results in dsRNA production. To block viral splicing we utilized antisense phosphorodiamidate morpholino oligomers (Morpholinos) that target specific splice donors or splice acceptors within viral pre-mRNA to preclude spliceosome access (72). We designed a pool of three Morpholinos that target only the top-strand late transcripts including the TPL exon 2 splice donor, the TPL exon 3 splice donor, and the downstream Fiber splice acceptor. We observed a significant decrease in splice efficiency of viral late transcripts, with no impact on a non-targeted viral early transcript (Figure 9A). When we delivered these Morpholinos immediately after infection and assayed for the production of dsRNA by IFA, we were now able to see dsRNA produced in WT Ad5-infected cells (Figure 9B, C). Taken together, these data highlight the process of efficient splicing as a strategy by which double-stranded DNA viruses avoid the production of deleterious dsRNA and activation of the innate immune system.

## DISCUSSION

### A novel mechanism for preventing the production of dsRNA

**Figure 10.**
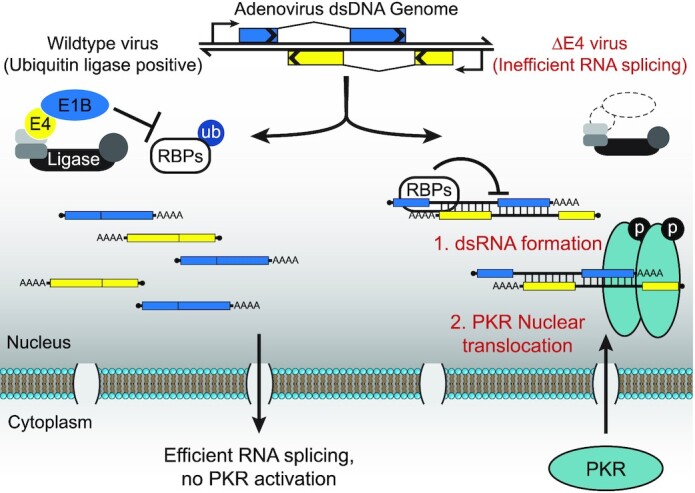
Model depicting how inefficient splicing of overlapping viral transcripts can lead to intermolecular dsRNA formation. During wildtype virus infection the presence of the E1B55K/E4orf6 viral hijacked ubiquitin ligase leads to the ubiquitination of cellular RNA binding proteins (RBPs) RALY and hnRNPC, which precludes their binding to viral RNA. In the presence of RALY and hnRNPC viral transcripts are poorly spliced, leading to the formation of dsRNA between the exonic regions of viral RNAs to the intronic regions of transcripts derived from the opposing strand. After these dsRNA molecules form, a fraction of the cytoplasmic dsRNA-sensor PKR translocates into the nucleus, where it co-localizes with viral dsRNA and is activated by auto-phosphorylation.

For decades it has been assumed that DNA viruses produce dsRNA during infection as a consequence of symmetrical transcription (21). While some early biochemical studies supported this hypothesis (35,73–75), technical challenges precluded definitive evidence for viral-derived dsRNA. Although a previous study observed dsRNA in fixed cells infected with wildtype AdV (76), these results have not been replicated in our study. Here we show that wildtype Ad5 does not produce detectable levels of dsRNA during infection. In contrast, AdV infections lacking virus-directed ubiquitination activity produce abundant nuclear dsRNA that is composed of unspliced or poorly processed viral transcripts. This viral dsRNA activates the cellular sensor PKR, even in the presence of the well-characterized viral PKR inhibitor VA RNA. Nuclear accumulation of dsRNA precedes the translocation of PKR from its typical cytoplasmic compartment to the nucleus. Ultimately, dsRNA production from mutant viruses can be rescued by removal of inhibitory cellular RNA binding proteins that otherwise impair efficient splicing. We propose that ubiquitination of these host RNA binding proteins during wildtype infection overcomes the splicing defects that would otherwise produce dsRNA. By maintaining a genome organization that has minimal overlap between long-lived exonic RNAs from top and bottom strands, and by actively modulating cellular splicing factors to promote efficient viral RNA splicing, adenovirus demonstrates a novel mechanism for preventing the production of dsRNA that would otherwise activate the innate immune system. Our findings are summarized in Figure 10.

### Could there be small amounts of dsRNA below the level of detection?

While we did not detect any evidence of viral dsRNA during WT Ad5 infection, it is impossible to rule out that small amounts of dsRNA molecules are produced during WT infection that are below the limit of detection by anti-dsRNA antibodies. Weber and colleagues were able to visualize nuclear and cytoplasmic dsRNA using the J2 monoclonal during infection with DNA viruses Ad5 as well as HSV-1 (76). Importantly, this group used a Cy3-based tyramide signal amplification system for immunofluorescence, yet still failed to see dsRNA production during negative-sense influenza or La Crosse virus infection. Subsequently, Son and colleagues characterized the 9D5 anti-dsRNA antibody as recognizing the same dsRNA structures (55), albeit at over 20 times the sensitivity (56). Using the 9D5 antibody, dsRNA was now readily detectable during infection with many diverse positive and negative sense RNA viruses including influenza A virus, vesicular stomatitis virus, measles virus, lymphocytic choriomeningitis virus, and arenavirus without the need for any signal amplification (55,56,77). Strikingly, we were not able to detect dsRNA using the 9D5 antibody during wildtype Ad5 infection, however we have not utilized any form of fluorescence signal amplification.

Given the difficulty in trying to prove a negative hypothesis, future studies should aim to use orthogonal methods to search for potentially very low levels of dsRNA during viral infection. In one strategy, a professional cellular dsRNA sensor (RIG-I) was *in vitro* purified and utilized to enrich viral RNA in a cross-linking and immunoprecipitation method (32). Alternatively, viral dsRNA-binding proteins have been purified and used as probes to detect dsRNA both *in vitro* and *in vivo* (78), and these could also be adapted for sequencing-based studies. A potential caveat of both techniques is that these dsRNA-binding proteins are also known to have low affinity for single-stranded RNA, complicating interpretation of any sequencing results (6). A similar approach exploits the oligomer-forming capability of MDA5 filaments to distinguish long strands of viral dsRNA, however these techniques are currently performed on extracted and purified RNA (79,80). Alternatively, one could use small molecule-based psoralen crosslinking and sequencing approaches to isolate double-stranded nucleic acids in living cells prior to sequencing (81–83). While often used to determine secondary structures of RNA molecules *in cis*, the strand-specific mapping of these sequencing approaches can be used to demonstrate intermolecular dsRNA *in trans* that is predicted to form during DNA virus infection.

### Why does adenovirus possess an antagonist of PKR?

An outstanding question remains as to why all human adenoviruses encode PKR-blocking virus associated RNAs if viral modulation of RNA processing is sufficient to impair dsRNA formation. One hypothesis is that VA RNAs act as a fail-safe for low-levels of dsRNA that might still form during WT infection. Alternatively, the ability of adenovirus to modulate cellular RNA processing might be dependent on the cell-type or cell-status of the infected cell. In this study, we have looked exclusively at cancer cells or immortalized cell lines *in vitro*. While the absence of dsRNA in the immortalized, but karyotypically stable, human bronchial epithelial cells argues against our observed phenotype being solely a cancer cell artifact (84), more investigation is warranted. In particular, the study of adenovirus infection in a living animal would be highly informative, although the lack of suitable models for human adenovirus makes this difficult (85). As an alternative, the use of highly stratified human organoids, coupled with serotypes of adenovirus that target different organ systems, might reveal scenarios where viral dsRNA is detected (86–88).

How PKR is activated during infection with ΔVA in the absence of detectable dsRNA production remains an unanswered question. As a master regulator of translation, PKR is also activated independently of dsRNA following diverse non-viral insults, including oxidative (89), endoplasmic reticulum (90), and metabolic stress (91). The precise mechanism of activation is unknown in many cases; however, several host and viral proteins and single-stranded RNAs have been shown to regulate PKR (14). Other dsRNA-binding proteins activate or inhibit PKR either through direct protein–protein interactions or through bridging interactions with or competition for dsRNA (92). VA RNA binds several host dsRNA-binding proteins in addition to PKR (93). Rather than outcompeting dsRNA, VA RNA may prevent AdV-induced PKR activation by outcompeting or mislocalizing a protein activator or by recruiting an inhibitor to PKR. PKR can also be activated by RNA secondary structures within the UTRs of host mRNAs or single-stranded RNA virus genomes (94–96). Of note, the 5′ UTR of all human AdV late mRNAs contains the highly structured TPL which is essential for protein translation. One study found VA RNA bound to AdV late transcripts and it could possibly act locally to prevent TPL-induced PKR activation (97). These non-canonical protein and RNA activators of PKR are understudied in the context of DNA virus infection and merit future investigation.

### Are there specific sensors for dsRNA in the nucleus?

There is growing appreciation for the role of endogenous dsRNA recognition in the fields of human health and autoimmunity (53,98–103). Typically, low-level endogenous dsRNA is produced in the nucleus and deaminated by the action of Adenosine Deaminase Acting on RNA (ADAR) proteins to convert adenosines to inosines (22). This RNA editing serves at least two purposes: inosine-modified dsRNA are often retained within subnuclear compartments called paraspeckles which preclude export into the cytoplasm and translation (104,105). In addition, modified RNAs have lower affinity for antiviral dsRNA sensors like RIG-I, MDA5, OASs or PKR (106,107). Thus, the majority of nuclear dsRNA might go unnoticed by the innate immune system in unstressed cells due to the absence of most canonical PRRs within this subcellular compartment (108,109). It is intriguing to speculate that viral infection itself might alter the host cell's ability to tolerate nuclear dsRNA. To our knowledge, our study is the first to show PKR translocation to the nucleus of cells expressing viral dsRNA. Intriguingly, while PKR was phosphorylated and activated in the nucleus, the downstream kinase target eIF2α remained in the cytoplasm during infection. The mechanism by which PKR translocates to the nucleus, and potentially shuttles to activate downstream signaling cascades, is of broad interest. It is known that the localization of many nuclear dsRNA binding proteins is affected by their interaction with RNA (110,111), and one could hypothesize that viral RNAs themselves might cause relocalization of these factors. Alternatively, the changing pool of cellular RNAs during viral infection, through upregulation of endogenous retroelements or dysregulation of ADAR editing (24,112,113), might lead to a redistribution of cellular antiviral sensors. It will be interesting to determine whether additional cytoplasmic sensors such as RLRs or OAS proteins are relocalized to the nucleus in the presence of nuclear dsRNA. Alternatively, one could hypothesize the existence of nuclear-resident dsRNA sensors that mediate downstream antiviral signaling or protein translocations. The mechanisms for cytoplasmic/nuclear trafficking of PKR and other dsRNA sensors during ΔE4 virus-infection may apply to diverse DNA viruses as well as nuclear-replicating RNA viruses such as influenza (111,114).

Compared to compact viral transcriptomes, cellular genes often contain an abundance of coding space to allow for unhindered RNA processing. As such, it would be curious to see whether poor RNA processing leading to dsRNA production is a problem unique to viral infection. Cell stresses such as osmotic shock or viral infection can cause polyadenylation readthrough which could lead to increased cellular dsRNA production from neighboring antisense transcripts (115–120). Recent studies have also implicated aberrant splicing in certain cancers as a potential therapeutic target (121,122). Of particular interest, when Bowling and Wang *et al.* treated *Myc*-driven triple-negative breast cancer cells with the spliceosome targeted therapy H3B-8800, or directly degraded the splicing factor SF3B1, they saw increased endogenous dsRNA production leading to inflammatory cell death through antiviral pathways (122). These results imply that the process of RNA splicing, besides its critical role in gene expression, serves as a powerful tool to regulate self versus non-self recognition. As a nuclear-replicating virus that is reliant on cellular RNA processing machinery, adenovirus is an exceptional model to address these topics.

## DATA AVAILABILITY

Fastq and mapped datasets generated from Illumina sequencing has been made publicly available at GEO (GSE179388). The authors declare that all other data supporting the findings of this study are available within the article and its Supplementary Information files, or are available from the authors upon request.

## Supplementary Material

gkab896_Supplemental_File

## Notes

Present address: Yize Li, The Biodesign Institute, Center for Immunotherapy, Vaccines and Virotherapy, School of Life Sciences, Arizona State University, Tempe, AZ, USA.

Present address: Christin Herrmann, Kimmel Center for Biology and Medicine at the Skirball Institute, New York University Grossman School of Medicine, New York, NY, USA.

## References

[1] Chan Y.K. , Gack M.U. Viral evasion of intracellular DNA and RNA sensing. Nat. Rev. Microbiol. 2016; 14:360–373. 27174148 10.1038/nrmicro.2016.45 PMC5072394

[2] Fensterl V. , Chattopadhyay S., Sen G.C. No love lost between viruses and interferons. Annu Rev Virol. 2015; 2:549–572. 26958928 10.1146/annurev-virology-100114-055249 PMC9549753

[3] Gebhardt A. , Laudenbach B.T., Pichlmair A. Discrimination of self and non-self ribonucleic acids. J. Interferon Cytokine Res. 2017; 37:184–197. 28475460 10.1089/jir.2016.0092 PMC5439445

[4] Unterholzner L. , Keating S.E., Baran M., Horan K.A., Jensen S.B., Sharma S., Sirois C.M., Jin T., Latz E., Xiao T.S. et al. IFI16 is an innate immune sensor for intracellular DNA. Nat. Immunol. 2010; 11:997–1004. 20890285 10.1038/ni.1932 PMC3142795

[5] Sun L. , Wu J., Du F., Chen X., Chen Z.J. Cyclic GMP-AMP synthase is a cytosolic DNA sensor that activates the type I interferon pathway. Science. 2013; 339:786–791. 23258413 10.1126/science.1232458 PMC3863629

[6] Hur S. Double-Stranded RNA sensors and modulators in innate immunity. Annu. Rev. Immunol. 2019; 37:349–375. 30673536 10.1146/annurev-immunol-042718-041356 PMC7136661

[7] Yoneyama M. , Onomoto K., Jogi M., Akaboshi T., Fujita T. Viral RNA detection by RIG-I-like receptors. Curr. Opin. Immunol. 2015; 32:48–53. 25594890 10.1016/j.coi.2014.12.012

[8] Zhao Y. , Karijolich J. Know Thyself: RIG-I-Like receptor sensing of DNA virus infection. J. Virol. 2019; 93:e01085-19. 31511389 10.1128/JVI.01085-19 PMC6854496

[9] Silverman R.H. Viral Encounters with 2′, 5′-oligoadenylate synthetase and RNase L during the interferon antiviral response. J. Virol. 2007; 81:12720–12729. 17804500 10.1128/JVI.01471-07 PMC2169107

[10] Silverman R.H. , Weiss S.R. Viral phosphodiesterases that antagonize double-stranded RNA signaling to RNase L by degrading 2-5A. J. Interferon Cytokine Res. 2014; 34:455–463. 24905202 10.1089/jir.2014.0007 PMC4046343

[11] Kristiansen H. , Gad H.H., Eskildsen-Larsen S., Despres P., Hartmann R. The oligoadenylate synthetase family: an ancient protein family with multiple antiviral activities. J. Interferon Cytokine Res. 2011; 31:41–47. 21142819 10.1089/jir.2010.0107

[12] Langland J.O. , Cameron J.M., Heck M.C., Jancovich J.K., Jacobs B.L. Inhibition of PKR by RNA and DNA viruses. Virus Res. 2006; 119:100–110. 16704884 10.1016/j.virusres.2005.10.014

[13] Sadler A.J. , Williams B.R. Structure and function of the protein kinase R. Curr. Top. Microbiol. Immunol. 2007; 316:253–292. 17969452 10.1007/978-3-540-71329-6_13

[14] Bou-Nader C. , Gordon J.M., Henderson F.E., Zhang J. The search for a PKR code—differential regulation of protein kinase R activity by diverse RNA and protein regulators. RNA. 2019; 25:539–556. 30770398 10.1261/rna.070169.118 PMC6467004

[15] Meyerson N.R. , Sawyer S.L. Two-stepping through time: mammals and viruses. Trends Microbiol. 2011; 19:286–294. 21531564 10.1016/j.tim.2011.03.006 PMC3567447

[16] Duggal N.K. , Emerman M. Evolutionary conflicts between viruses and restriction factors shape immunity. Nat. Rev. Immunol. 2012; 12:687–695. 22976433 10.1038/nri3295 PMC3690816

[17] Daugherty M.D. , Malik H.S. Rules of engagement: molecular insights from host-virus arms races. Annu. Rev. Genet. 2012; 46:677–700. 23145935 10.1146/annurev-genet-110711-155522

[18] Carpentier K.S. , Geballe A.P. An evolutionary view of the arms race between protein kinase R and large DNA viruses. J. Virol. 2016; 90:3280–3283. 26792736 10.1128/JVI.01996-15 PMC4794663

[19] Berk AJ. Knipe D.M. , Howley P.M. Adenoviridae. Fields Virology. 2013; 2:Philadelphia Wolters Kluwer Health/Lippincott Williams & Wilkins 1704–1731.

[20] Ghebremedhin B. Human adenovirus: viral pathogen with increasing importance. Eur. J. Microbiol. Immunol. (Bp). 2014; 4:26–33. 24678403 10.1556/EuJMI.4.2014.1.2 PMC3955829

[21] Jacobs B.L. , Langland J.O. When two strands are better than one: the mediators and modulators of the cellular responses to double-stranded RNA. Virology. 1996; 219:339–349. 8638399 10.1006/viro.1996.0259

[22] George C.X. , John L., Samuel C.E. An RNA editor, adenosine deaminase acting on double-stranded RNA (ADAR1). J. Interferon Cytokine Res. 2014; 34:437–446. 24905200 10.1089/jir.2014.0001 PMC4046350

[23] Panning B. , Smiley J.R. Activation of RNA polymerase III transcription of human Alu repetitive elements by adenovirus type 5: requirement for the E1b 58-kilodalton protein and the products of E4 open reading frames 3 and 6. Mol. Cell. Biol. 1993; 13:3231–3244. 7684492 10.1128/mcb.13.6.3231-3244.1993 PMC359768

[24] Panning B. , Smiley J.R. Activation of expression of multiple subfamilies of human Alu elements by adenovirus type 5 and herpes simplex virus type 1. J. Mol. Biol. 1995; 248:513–524. 7752221 10.1006/jmbi.1995.0239

[25] Russanova V.R. , Driscoll C.T., Howard B.H. Adenovirus type 2 preferentially stimulates polymerase III transcription of Alu elements by relieving repression: a potential role for chromatin. Mol. Cell. Biol. 1995; 15:4282–4290. 7623822 10.1128/mcb.15.8.4282 PMC230667

[26] Dunker W. , Zhao Y., Song Y., Karijolich J. Recognizing the SINEs of Infection: Regulation of retrotransposon expression and modulation of host cell processes. Viruses. 2017; 9:386. 10.3390/v9120386 PMC5744160 29258254

[27] Burgess H.M. , Mohr I. Cellular 5′-3′ mRNA exonuclease Xrn1 controls double-stranded RNA accumulation and anti-viral responses. Cell Host Microbe. 2015; 17:332–344. 25766294 10.1016/j.chom.2015.02.003 PMC4826345

[28] Liu S.-W. , Katsafanas G.C., Liu R., Wyatt L.S., Moss B. Poxvirus decapping enzymes enhance virulence by preventing the accumulation of dsRNA and the induction of innate antiviral responses. Cell Host Microbe. 2015; 17:320–331. 25766293 10.1016/j.chom.2015.02.002 PMC4359750

[29] Burgess H.M. , Mohr I. Defining the role of stress granules in innate immune suppression by the herpes simplex virus 1 endoribonuclease VHS. J. Virol. 2018; 92:e00829-18. 29793959 10.1128/JVI.00829-18 PMC6052315

[30] Dauber B. , Saffran H.A., Smiley J.R. The herpes simplex virus host shutoff (vhs) RNase limits accumulation of double stranded RNA in infected cells: Evidence for accelerated decay of duplex RNA. PLoS Pathog. 2019; 15:e1008111. 31626661 10.1371/journal.ppat.1008111 PMC6821131

[31] West J.A. , Wicks M., Gregory S.M., Chugh P., Jacobs S.R., Zhang Z., Host K.M., Dittmer D.P., Damania B. An important role for mitochondrial antiviral signaling protein in the kaposi's sarcoma-associated herpesvirus life cycle. J. Virol. 2014; 88:5778–5787. 24623417 10.1128/JVI.03226-13 PMC4019080

[32] Zhang Y. , Dittmer D.P., Mieczkowski P.A., Host K.M., Fusco W.G., Duncan J.A., Damania B. RIG-I detects Kaposi's sarcoma-associated herpesvirus transcripts in a RNA polymerase III-independent manner. mBio. 2018; 9:e00823-18. 29970461 10.1128/mBio.00823-18 PMC6030556

[33] Zhao Y. , Ye X., Dunker W., Song Y., Karijolich J. RIG-I like receptor sensing of host RNAs facilitates the cell-intrinsic immune response to KSHV infection. Nat. Commun. 2018; 9:4841. 30451863 10.1038/s41467-018-07314-7 PMC6242832

[34] Herrmann C. , Dybas J.M., Liddle J.C., Price A.M., Hayer K.E., Lauman R., Purman C.E., Charman M., Kim E.T., Garcia B.A. et al. Adenovirus-mediated ubiquitination alters protein–RNA binding and aids viral RNA processing. Nature Microbiology. 2020; 5:1217–1231. 10.1038/s41564-020-0750-9 PMC7529849 32661314

[35] Maran A. , Mathews M.B. Characterization of the double-stranded RNA implicated in the inhibition of protein synthesis in cells infected with a mutant adenovirus defective for VA RNA. Virology. 1988; 164:106–113. 3363861 10.1016/0042-6822(88)90625-3

[36] Vachon V.K. , Conn G.L. Adenovirus VA RNA: an essential pro-viral non-coding RNA. Virus Res. 2016; 212:39–52. 26116898 10.1016/j.virusres.2015.06.018

[37] Hood I.V. , Gordon J.M., Bou-Nader C., Henderson F.E., Bahmanjah S., Zhang J. Crystal structure of an adenovirus virus-associated RNA. Nat. Commun. 2019; 10:2871. 31253805 10.1038/s41467-019-10752-6 PMC6599070

[38] Meng H. , Deo S., Xiong S., Dzananovic E., Donald L.J., van Dijk C.W., McKenna S.A. Regulation of the interferon-inducible 2′-5′-oligoadenylate synthetases by adenovirus VA(I) RNA. J. Mol. Biol. 2012; 422:635–649. 22709583 10.1016/j.jmb.2012.06.017

[39] Bridge E. , Ketner G. Interaction of adenoviral E4 and E1b products in late gene expression. Virology. 1990; 174:345–353. 2137659 10.1016/0042-6822(90)90088-9

[40] Bridge E. , Ketner G. Redundant control of adenovirus late gene expression by early region 4. J. Virol. 1989; 63:631–638. 2911117 10.1128/jvi.63.2.631-638.1989 PMC247733

[41] Halbert D.N. , Cutt J.R., Shenk T. Adenovirus early region 4 encodes functions required for efficient DNA replication, late gene expression, and host cell shutoff. J. Virol. 1985; 56:250–257. 4032537 10.1128/jvi.56.1.250-257.1985 PMC252513

[42] Huang M.M. , Hearing P. Adenovirus early region 4 encodes two gene products with redundant effects in lytic infection. J. Virol. 1989; 63:2605–2615. 2724411 10.1128/jvi.63.6.2605-2615.1989 PMC250738

[43] Bhat R.A. , Thimmappaya B. Adenovirus mutants with DNA sequence perturbations in the intragenic promoter of VAI RNA gene allow the enhanced transcription of VAII RNA gene in HeLa cells. Nucleic Acids Res. 1984; 12:7377–7388. 6493978 10.1093/nar/12.19.7377 PMC320168

[44] Weinberg D.H. , Ketner G. A cell line that supports the growth of a defective early region 4 deletion mutant of human adenovirus type 2. Proc. Natl. Acad. Sci. U.S.A. 1983; 80:5383–5386. 6310575 10.1073/pnas.80.17.5383 PMC384260

[45] Li Y. , Banerjee S., Goldstein S.A., Dong B., Gaughan C., Rath S., Donovan J., Korennykh A., Silverman R.H., Weiss S.R. Ribonuclease L mediates the cell-lethal phenotype of double-stranded RNA editing enzyme ADAR1 deficiency in a human cell line. Elife. 2017; 6:e25687. 28362255 10.7554/eLife.25687 PMC5404912

[46] Wang T. , Wei J.J., Sabatini D.M., Lander E.S. Genetic screens in human cells using the CRISPR-Cas9 System. Science. 2014; 343:80–84. 24336569 10.1126/science.1246981 PMC3972032

[47] Ran F.A. , Hsu P.D., Wright J., Agarwala V., Scott D.A., Zhang F. Genome engineering using the CRISPR-Cas9 system. Nat. Protoc. 2013; 8:2281–2308. 24157548 10.1038/nprot.2013.143 PMC3969860

[48] Suzuki K. , Bose P., Leong-Quong R.Y., Fujita D.J., Riabowol K. REAP: a two minute cell fractionation method. BMC Research Notes. 2010; 3:294. 21067583 10.1186/1756-0500-3-294 PMC2993727

[49] Karijolich J. , Zhao Y., Alla R., Glaunsinger B. Genome-wide mapping of infection-induced SINE RNAs reveals a role in selective mRNA export. Nucleic Acids Res. 2017; 45:6194–6208. 28334904 10.1093/nar/gkx180 PMC5449642

[50] Decker C.J. , Steiner H.R., Hoon-Hanks L.L., Morrison J.H., Haist K.C., Stabell A.C., Poeschla E.M., Morrison T.E., Stenglein M.D., Sawyer S.L. et al. dsRNA-Seq: Identification of viral infection by purifying and sequencing dsRNA. Viruses. 2019; 11:943. 10.3390/v11100943 PMC6832592 31615058

[51] Price A.M. , Hayer K.E., Depledge D.P., Wilson A.C., Weitzman M.D. Novel splicing and open reading frames revealed by long-read direct RNA sequencing of adenovirus transcripts. 2019; bioRxiv doi:13 December 2019, preprint: not peer reviewed 10.1101/2019.12.13.876037. PMC9499273 36095031

[52] Schönborn J. , Oberstrass J., Breyel E., Tittgen J., Schumacher J., Lukacs N. Monoclonal antibodies to double-stranded RNA as probes of RNA structure in crude nucleic acid extracts. Nucleic Acids Res. 1991; 19:2993–3000. 2057357 10.1093/nar/19.11.2993 PMC328262

[53] Dhir A. , Dhir S., Borowski L.S., Jimenez L., Teitell M., Rotig A., Crow Y.J., Rice G.I., Duffy D., Tamby C. et al. Mitochondrial double-stranded RNA triggers antiviral signalling in humans. Nature. 2018; 560:238–242. 30046113 10.1038/s41586-018-0363-0 PMC6570621

[54] Sandler A.B. , Ketner G. Adenovirus early region 4 is essential for normal stability of late nuclear RNAs. J. Virol. 1989; 63:624–630. 2911116 10.1128/jvi.63.2.624-630.1989 PMC247732

[55] Son K.N. , Liang Z., Lipton H.L. Double-stranded RNA is detected by immunofluorescence analysis in RNA and DNA virus infections, including those by negative-stranded RNA viruses. J. Virol. 2015; 89:9383–9392. 26136565 10.1128/JVI.01299-15 PMC4542381

[56] Mateer E.J. , Paessler S., Huang C. Visualization of Double-Stranded RNA colocalizing with pattern recognition receptors in arenavirus infected cells. Front. Cell. Infect. Microbiol. 2018; 8:e251. 10.3389/fcimb.2018.00251 PMC6066581 30087859

[57] Querido E. , Marcellus R.C., Lai A., Charbonneau R., Teodoro J.G., Ketner G., Branton P.E. Regulation of p53 levels by the E1B 55-kilodalton protein and E4orf6 in adenovirus-infected cells. J. Virol. 1997; 71:3788–3798. 9094654 10.1128/jvi.71.5.3788-3798.1997 PMC191529

[58] Stracker T.H. , Carson C.T., Weitzman M.D. Adenovirus oncoproteins inactivate the Mre11-Rad50-NBS1 DNA repair complex. Nature. 2002; 418:348–352. 12124628 10.1038/nature00863

[59] Weitzman M.D. , Ornelles D.A. Inactivating intracellular antiviral responses during adenovirus infection. Oncogene. 2005; 24:7686–7696. 16299529 10.1038/sj.onc.1209063

[60] Kleinberger T. Biology of the adenovirus E4orf4 protein: from virus infection to cancer cell death. FEBS Lett. 2020; 594:1891–1917. 31792953 10.1002/1873-3468.13704

[61] Cathomen T. , Weitzman M.D. A functional complex of adenovirus proteins E1B-55kDa and E4orf6 is necessary to modulate the expression level of p53 but not its transcriptional activity. J. Virol. 2000; 74:11407–11412. 11070042 10.1128/jvi.74.23.11407-11412.2000 PMC113247

[62] Graham F.L. , Smiley J., Russell W.C., Nairn R. Characteristics of a human cell line transformed by DNA from human adenovirus type 5. J. Gen. Virol. 1977; 36:59–74. 886304 10.1099/0022-1317-36-1-59

[63] Soucy T.A. , Smith P.G., Milhollen M.A., Berger A.J., Gavin J.M., Adhikari S., Brownell J.E., Burke K.E., Cardin D.P., Critchley S. et al. An inhibitor of NEDD8-activating enzyme as a new approach to treat cancer. Nature. 2009; 458:732–736. 19360080 10.1038/nature07884

[64] Jones J. , Wu K., Yang Y., Guerrero C., Nillegoda N., Pan Z.-Q., Huang L. A targeted proteomic analysis of the ubiquitin-like modifier Nedd8 and associated proteins. J. Proteome Res. 2008; 7:1274–1287. 18247557 10.1021/pr700749v PMC2676899

[65] Forrester N.A. , Sedgwick G.G., Thomas A., Blackford A.N., Speiseder T., Dobner T., Byrd P.J., Stewart G.S., Turnell A.S., Grand R.J.A. Serotype-specific inactivation of the cellular DNA damage response during adenovirus infection. J. Virol. 2011; 85:2201–2211. 21159879 10.1128/JVI.01748-10 PMC3067775

[66] Spurgeon M.E. , Ornelles D.A. The adenovirus E1B 55-kilodalton and E4 open reading frame 6 proteins limit phosphorylation of eIF2alpha during the late phase of infection. J. Virol. 2009; 83:9970–9982. 19605483 10.1128/JVI.01113-09 PMC2747998

[67] O’Malley R.P. , Mariano T.M., Siekierka J., Mathews M.B. A mechanism for the control of protein synthesis by adenovirus VA RNAI. Cell. 1986; 44:391–400. 3943131 10.1016/0092-8674(86)90460-5

[68] Kamel W. , Segerman B., Öberg D., Punga T., Akusjärvi G. The adenovirus VA RNA-derived miRNAs are not essential for lytic virus growth in tissue culture cells. Nucleic Acids Res. 2013; 41:4802–4812. 23525465 10.1093/nar/gkt172 PMC3643585

[69] Pombo A. , Ferreira J., Bridge E., Carmo-Fonseca M. Adenovirus replication and transcription sites are spatially separated in the nucleus of infected cells. EMBO J. 1994; 13:5075–5085. 7957073 10.1002/j.1460-2075.1994.tb06837.x PMC395454

[70] Price A.M. , Hayer K.E., McIntyre A.B.R., Gokhale N.S., Abebe J.S., Della Fera A.N., Mason C.E., Horner S.M., Wilson A.C., Depledge D.P. et al. Direct RNA sequencing reveals m 6 A modifications on adenovirus RNA are necessary for efficient splicing. Nat. Commun. 2020; 11:6016. 33243990 10.1038/s41467-020-19787-6 PMC7691994

[71] Kim Y. , Park J., Kim S., Kim M., Kang M.-G., Kwak C., Kang M., Kim B., Rhee H.-W., Kim V.N. PKR senses nuclear and mitochondrial signals by interacting with endogenous Double-Stranded RNAs. Mol. Cell. 2018; 71:1051–1063. 30174290 10.1016/j.molcel.2018.07.029

[72] Morcos P.A. Achieving targeted and quantifiable alteration of mRNA splicing with Morpholino oligos. Biochem. Biophys. Res. Commun. 2007; 358:521–527. 17493584 10.1016/j.bbrc.2007.04.172

[73] Jacquemont B. , Roizman B. RNA synthesis in cells infected with herpes simplex virus. X. Properties of viral symmetric transcripts and of double-stranded RNA prepared from them. J. Virol. 1975; 15:707–713. 163916 10.1128/jvi.15.4.707-713.1975 PMC354512

[74] Aloni Y. Extensive symmetrical transcription of Simian Virus 40 DNA in virus-yielding cells. Proc. Natl. Acad. Sci. U.S.A. 1972; 69:2404–2409. 4341693 10.1073/pnas.69.9.2404 PMC426950

[75] Boone R.F. , Parr R.P., Moss B. Intermolecular duplexes formed from polyadenylylated vaccinia virus RNA. J. Virol. 1979; 30:365–374. 480457 10.1128/jvi.30.1.365-374.1979 PMC353330

[76] Weber F. , Wagner V., Rasmussen S.B., Hartmann R., Paludan S.R. Double-stranded RNA is produced by positive-strand RNA viruses and DNA viruses but not in detectable amounts by negative-strand RNA viruses. J. Virol. 2006; 80:5059–5064. 16641297 10.1128/JVI.80.10.5059-5064.2006 PMC1472073

[77] Pfaller C.K. , Mastorakos G.M., Matchett W.E., Ma X., Samuel C.E., Cattaneo R. Measles virus defective interfering RNAs are generated frequently and early in the absence of C protein and can be destabilized by adenosine deaminase acting on RNA-1-Like hypermutations. J. Virol. 2015; 89:7735–7747. 25972541 10.1128/JVI.01017-15 PMC4505647

[78] Monsion B. , Incarbone M., Hleibieh K., Poignavent V., Ghannam A., Dunoyer P., Daeffler L., Tilsner J., Ritzenthaler C. Efficient detection of long dsRNA in vitro and in vivo using the dsRNA binding domain from FHV B2 protein. Front Plant Sci. 2018; 9:70. 29449856 10.3389/fpls.2018.00070 PMC5799278

[79] Peisley A. , Jo M.H., Lin C., Wu B., Orme-Johnson M., Walz T., Hohng S., Hur S. Kinetic mechanism for viral dsRNA length discrimination by MDA5 filaments. Proc. Natl. Acad. Sci. U.S.A. 2012; 109:E3340–E3349. 23129641 10.1073/pnas.1208618109 PMC3523859

[80] Wu B. , Huoh Y.-S., Hur S. Measuring monomer-to-filament transition of MAVS as an in vitro activity assay for RIG-I-Like receptors. Methods Mol. Biol. 2016; 1390:131–142. 26803627 10.1007/978-1-4939-3335-8_9 PMC6122957

[81] Lu Z. , Zhang Q.C., Lee B., Flynn R.A., Smith M.A., Robinson J.T., Davidovich C., Gooding A.R., Goodrich K.J., Mattick J.S. et al. RNA duplex map in living cells reveals higher-order transcriptome structure. Cell. 2016; 165:1267–1279. 27180905 10.1016/j.cell.2016.04.028 PMC5029792

[82] Sharma E. , Sterne-Weiler T., O’Hanlon D., Blencowe B.J. Global mapping of human RNA-RNA interactions. Mol. Cell. 2016; 62:618–626. 27184080 10.1016/j.molcel.2016.04.030

[83] Ziv O. , Gabryelska M.M., Lun A.T.L., Gebert L.F.R., Sheu-Gruttadauria J., Meredith L.W., Liu Z.-Y., Kwok C.K., Qin C.-F., MacRae I.J. et al. COMRADES determines in vivo RNA structures and interactions. Nat. Methods. 2018; 15:785–788. 30202058 10.1038/s41592-018-0121-0 PMC6168409

[84] Ramirez R.D. , Sheridan S., Girard L., Sato M., Kim Y., Pollack J., Peyton M., Zou Y., Kurie J.M., Dimaio J.M. et al. Immortalization of human bronchial epithelial cells in the absence of viral oncoproteins. Cancer Res. 2004; 64:9027–9034. 15604268 10.1158/0008-5472.CAN-04-3703

[85] Rodríguez E. , Ip W.H., Kolbe V., Hartmann K., Pilnitz-Stolze G., Tekin N., Gómez-Medina S., Muñoz-Fontela C., Krasemann S., Dobner T. Humanized mice reproduce acute and persistent human adenovirus infection. J. Infect. Dis. 2017; 215:70–79. 28077585 10.1093/infdis/jiw499

[86] Holly M.K. , Smith J.G. Adenovirus infection of human enteroids reveals interferon sensitivity and preferential infection of goblet cells. J. Virol. 2018; 92:e00250-18. 29467318 10.1128/JVI.00250-18 PMC5899204

[87] Kolawole A.O. , Wobus C.E. Gastrointestinal organoid technology advances studies of enteric virus biology. PLoS Pathog. 2020; 16:e1008212. 31999791 10.1371/journal.ppat.1008212 PMC6991956

[88] Porotto M. , Ferren M., Chen Y.-W., Siu Y., Makhsous N., Rima B., Briese T., Greninger A.L., Snoeck H.-W., Moscona A. Authentic modeling of human respiratory virus infection in human pluripotent stem Cell-Derived lung organoids. mBio. 2019; 10:e00723-19. 31064833 10.1128/mBio.00723-19 PMC6509192

[89] Patel R.C. , Sen G.C. PACT, a protein activator of the interferon-induced protein kinase, PKR. EMBO J. 1998; 17:4379–4390. 9687506 10.1093/emboj/17.15.4379 PMC1170771

[90] Lee E.-S. , Yoon C.-H., Kim Y.-S., Bae Y.-S. The double-strand RNA-dependent protein kinase PKR plays a significant role in a sustained ER stress-induced apoptosis. FEBS Lett. 2007; 581:4325–4332. 17716668 10.1016/j.febslet.2007.08.001

[91] Nakamura T. , Kunz R.C., Zhang C., Kimura T., Yuan C.L., Baccaro B., Namiki Y., Gygi S.P., Hotamisligil G.S. A critical role for PKR complexes with TRBP in Immunometabolic regulation and eIF2α phosphorylation in obesity. Cell Rep. 2015; 11:295–307. 25843719 10.1016/j.celrep.2015.03.021 PMC4439210

[92] Chang K.-Y. , Ramos A. The double-stranded RNA-binding motif, a versatile macromolecular docking platform. FEBS J. 2005; 272:2109–2117. 15853796 10.1111/j.1742-4658.2005.04652.x

[93] Liao H.J. , Kobayashi R., Mathews M.B. Activities of adenovirus virus-associated RNAs: purification and characterization of RNA binding proteins. Proc. Natl. Acad. Sci. U.S.A. 1998; 95:8514–8519. 9671709 10.1073/pnas.95.15.8514 PMC21107

[94] Cohen-Chalamish S. , Hasson A., Weinberg D., Namer L.S., Banai Y., Osman F., Kaempfer R. Dynamic refolding of IFN-gamma mRNA enables it to function as PKR activator and translation template. Nat. Chem. Biol. 2009; 5:896–903. 19801993 10.1038/nchembio.234

[95] Namer L.S. , Osman F., Banai Y., Masquida B., Jung R., Kaempfer R. An ancient pseudoknot in TNF-α pre-mRNA activates PKR, inducing eIF2α phosphorylation that potently enhances splicing. Cell Rep. 2017; 20:188–200. 28683312 10.1016/j.celrep.2017.06.035

[96] Toroney R. , Nallagatla S.R., Boyer J.A., Cameron C.E., Bevilacqua P.C. Regulation of PKR by HCV IRES RNA: importance of domain II and NS5A. J. Mol. Biol. 2010; 400:393–412. 20447405 10.1016/j.jmb.2010.04.059 PMC2902579

[97] Mathews M.B. Binding of adenovirus VA RNA to mRNA: a possible role in splicing. Nature. 1980; 285:575–577. 7402301 10.1038/285575a0

[98] Pajak A. , Laine I., Clemente P., El-Fissi N., Schober F.A., Maffezzini C., Calvo-Garrido J., Wibom R., Filograna R., Dhir A. et al. Defects of mitochondrial RNA turnover lead to the accumulation of double-stranded RNA in vivo. PLos Genet. 2019; 15:e1008240. 31365523 10.1371/journal.pgen.1008240 PMC6668790

[99] Rice G.I. , Kasher P.R., Forte G.M.A., Mannion N.M., Greenwood S.M., Szynkiewicz M., Dickerson J.E., Bhaskar S.S., Zampini M., Briggs T.A. et al. Mutations in ADAR1 cause Aicardi-Goutières syndrome associated with a type I interferon signature. Nat. Genet. 2012; 44:1243–1248. 23001123 10.1038/ng.2414 PMC4154508

[100] Chung H. , Calis J.J.A., Wu X., Sun T., Yu Y., Sarbanes S.L., Thi V.L.D., Shilvock A.R., Hoffmann H.-H., Rosenberg B.R. et al. Human ADAR1 prevents endogenous RNA from triggering translational shutdown. Cell. 2018; 172:811–824. 29395325 10.1016/j.cell.2017.12.038 PMC5831367

[101] Ahmad S. , Mu X., Yang F., Greenwald E., Park J.W., Jacob E., Zhang C.-Z., Hur S. Breaching self-tolerance to alu duplex RNA underlies MDA5-mediated inflammation. Cell. 2018; 172:797–810. 29395326 10.1016/j.cell.2017.12.016 PMC5807104

[102] Wolin S.L. , Maquat L.E. Cellular RNA surveillance in health and disease. Science. 2019; 366:822–827. 31727827 10.1126/science.aax2957 PMC6938259

[103] Liu G. , Gack M.U. Distinct and orchestrated functions of RNA Sensors in innate immunity. Immunity. 2020; 53:26–42. 32668226 10.1016/j.immuni.2020.03.017 PMC7367493

[104] Kumar M. , Carmichael G.G. Nuclear antisense RNA induces extensive adenosine modifications and nuclear retention of target transcripts. Proc. Natl. Acad. Sci. U.S.A. 1997; 94:3542–3547. 9108012 10.1073/pnas.94.8.3542 PMC20475

[105] Zhang Z. , Carmichael G.G. The fate of dsRNA in the nucleus: a p54nrb-containing complex mediates the nuclear retention of promiscuously A-to-I edited RNAs. Cell. 2001; 106:465–476. 11525732 10.1016/s0092-8674(01)00466-4

[106] Liddicoat B.J. , Piskol R., Chalk A.M., Ramaswami G., Higuchi M., Hartner J.C., Li J.B., Seeburg P.H., Walkley C.R. RNA editing by ADAR1 prevents MDA5 sensing of endogenous dsRNA as nonself. Science. 2015; 349:1115–1120. 26275108 10.1126/science.aac7049 PMC5444807

[107] Mannion N.M. , Greenwood S.M., Young R., Cox S., Brindle J., Read D., Nellaker C., Vesely C., Ponting C.P., McLaughlin P.J. et al. The RNA-editing enzyme ADAR1 controls innate immune responses to RNA. Cell Rep. 2014; 9:1482–1494. 25456137 10.1016/j.celrep.2014.10.041 PMC4542304

[108] Wilkins C. , Gale M. Recognition of viruses by cytoplasmic sensors. Curr. Opin. Immunol. 2010; 22:41–47. 20061127 10.1016/j.coi.2009.12.003 PMC3172156

[109] Habjan M. , Pichlmair A. Cytoplasmic sensing of viral nucleic acids. Curr. Opin. Virol. 2015; 11:31–37. 25668758 10.1016/j.coviro.2015.01.012 PMC7172233

[110] Fritz J. , Strehblow A., Taschner A., Schopoff S., Pasierbek P., Jantsch M.F. RNA-regulated interaction of transportin-1 and exportin-5 with the double-stranded RNA-binding domain regulates nucleocytoplasmic shuttling of ADAR1. Mol. Cell. Biol. 2009; 29:1487–1497. 19124606 10.1128/MCB.01519-08 PMC2648229

[111] Banerjee S. , Barraud P. Functions of double-stranded RNA-binding domains in nucleocytoplasmic transport. RNA Biol. 2014; 11:1226–1232. 25584639 10.4161/15476286.2014.972856 PMC4615638

[112] Samuel C.E. Adenosine deaminases acting on RNA (ADARs) are both antiviral and proviral. Virology. 2011; 411:180–193. 21211811 10.1016/j.virol.2010.12.004 PMC3057271

[113] Zhang H. , Ni G., Damania B. ADAR1 facilitates KSHV Lytic reactivation by modulating the RLR-Dependent signaling pathway. Cell Rep. 2020; 31:107564. 32348766 10.1016/j.celrep.2020.107564 PMC7319254

[114] Yarbrough M.L. , Mata M.A., Sakthivel R., Fontoura B.M. Viral subversion of nucleocytoplasmic trafficking. Traffic. 2014; 15:127–140. 24289861 10.1111/tra.12137 PMC3910510

[115] Rutkowski A.J. , Erhard F., L’Hernault A., Bonfert T., Schilhabel M., Crump C., Rosenstiel P., Efstathiou S., Zimmer R., Friedel C.C. et al. Widespread disruption of host transcription termination in HSV-1 infection. Nat. Commun. 2015; 6:7126. 25989971 10.1038/ncomms8126 PMC4441252

[116] Wyler E. , Menegatti J., Franke V., Kocks C., Boltengagen A., Hennig T., Theil K., Rutkowski A., Ferrai C., Baer L. et al. Widespread activation of antisense transcription of the host genome during herpes simplex virus 1 infection. Genome Biol. 2017; 18:209. 29089033 10.1186/s13059-017-1329-5 PMC5663069

[117] Hennig T. , Michalski M., Rutkowski A.J., Djakovic L., Whisnant A.W., Friedl M.S., Jha B.A., Baptista M.A.P., L’Hernault A., Erhard F. et al. HSV-1-induced disruption of transcription termination resembles a cellular stress response but selectively increases chromatin accessibility downstream of genes. PLoS Pathog. 2018; 14:e1006954. 29579120 10.1371/journal.ppat.1006954 PMC5886697

[118] Vilborg A. , Sabath N., Wiesel Y., Nathans J., Levy-Adam F., Yario T.A., Steitz J.A., Shalgi R. Comparative analysis reveals genomic features of stress-induced transcriptional readthrough. Proc. Natl Acad. Sci. U.S.A. 2017; 114:E8362–E8371. 28928151 10.1073/pnas.1711120114 PMC5635911

[119] Rosa-Mercado N.A. , Zimmer J.T., Apostolidi M., Rinehart J., Simon M.D., Steitz J.A. Hyperosmotic stress alters the RNA polymerase II interactome and induces readthrough transcription despite widespread transcriptional repression. Mol. Cell. 2021; 81:502–513. 33400923 10.1016/j.molcel.2020.12.002 PMC7867636

[120] Zinad H.S. , Natasya I., Werner A. Natural antisense transcripts at the interface between host genome and mobile genetic elements. Front. Microbiol. 2017; 8:2292. 29209299 10.3389/fmicb.2017.02292 PMC5701935

[121] Seiler M. , Yoshimi A., Darman R., Chan B., Keaney G., Thomas M., Agrawal A.A., Caleb B., Csibi A., Sean E. et al. H3B-8800, an orally available small-molecule splicing modulator, induces lethality in spliceosome-mutant cancers. Nat. Med. 2018; 24:497–504. 29457796 10.1038/nm.4493 PMC6730556

[122] Bowling E.A. , Wang J.H., Gong F., Wu W., Neill N.J., Kim I.S., Tyagi S., Orellana M., Kurley S.J., Dominguez-Vidaña R. et al. Spliceosome-targeted therapies trigger an antiviral immune response in triple-negative breast cancer. Cell. 2021; 184:384–403. 33450205 10.1016/j.cell.2020.12.031 PMC8635244

