# Supplementary material for: Adenovirus prevents dsRNA formation by promoting efficient splicing of viral RNA
Source: Nucleic Acids Res. 2021 Oct 21;50(3):1201–20. doi: 10.1093/nar/gkab896 (PMC8860579; doi:10.1093/nar/gkab896)
Supplement: gkab896_Supplemental_File [file gkab896_supplemental_file.pdf]

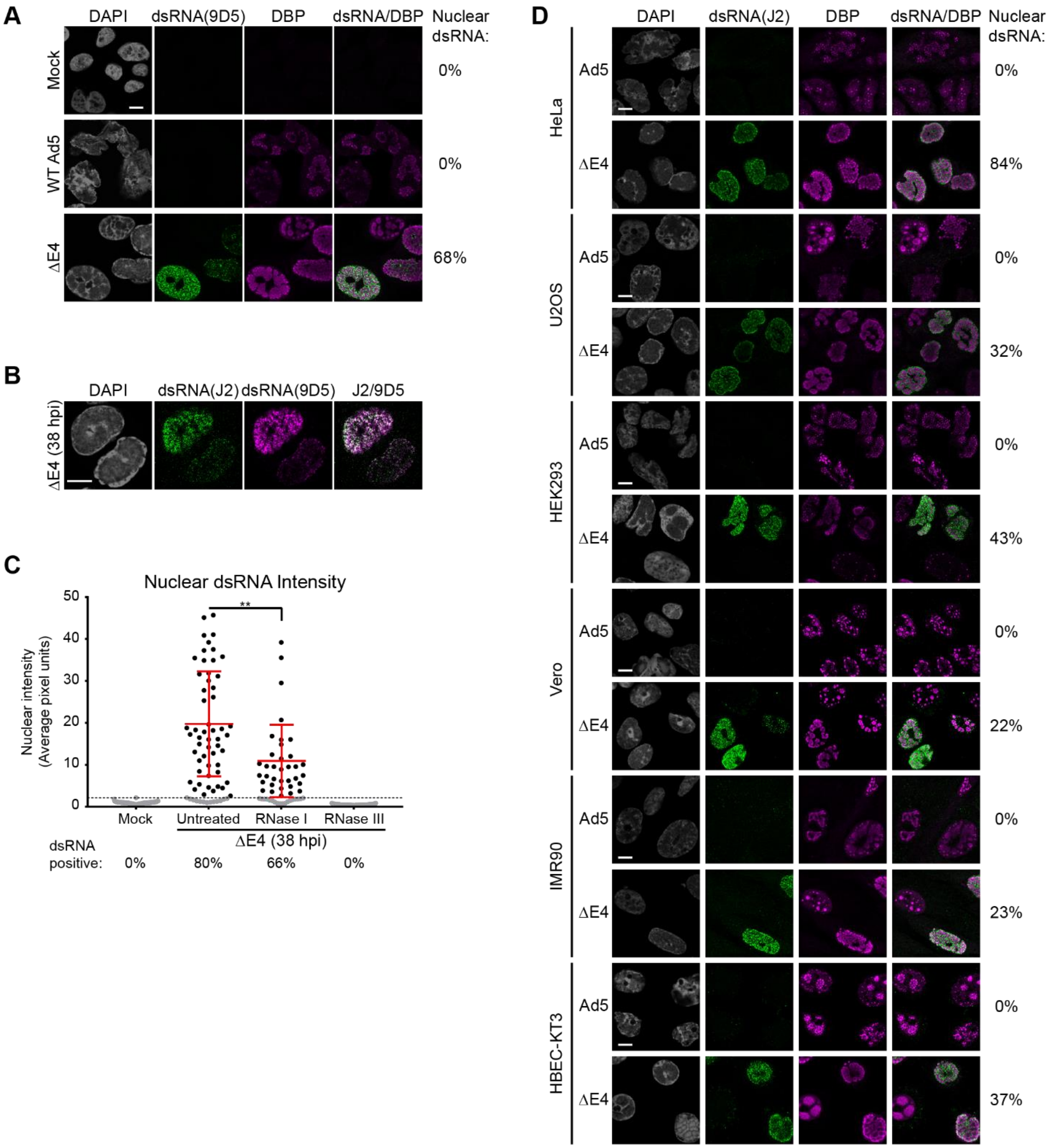

Supplementary Figure 1. Lack of WT adenovirus dsRNA production is not cell-type or antibody-dependent. (A) A549 cells were mock-, WT Ad5-, or ΔE4-infected for 48 hours and then stained with alternative α-dsRNA antibody (9D5, green) or VRC marker DBP (magenta). The percentage of all cells displaying nuclear dsRNA is shown to the right of each set. **(B)** J2 (green) and 9D5 (magenta) dsRNA antibodies stain the same foci within ΔE4-infected A549 cells. **(C)** A549 cells were infected with ΔE4 virus for 38 hours before fixation and permeabilization. Cells were treated for 30 minutes with single-stranded RNase (RNase I) or double-stranded RNase (RNase III) at 37^{0}C before being stained against dsRNA. Nuclear dsRNA was quantified as MNFI

and every cell displayed as a dot. The dsRNA positive threshold was defined as 4 standard deviations over the MNFI of dsRNA in uninfected cells and displayed as a dashed line. Cells below the threshold were colored grey, while cells positive for nuclear dsRNA were colored black and the mean and standard deviation of these positive cells is denoted by red error bars. The percentage of total cells expressing dsRNA during infection with each virus is shown below their name on the x-axis. **(D)** Multiple cell lines were infected with WT Ad5 or ΔE4 for 48 hours and stained with J2 (green) or VRC marker DBP (magenta). The percentage of all cells displaying nuclear dsRNA is shown to the right of each set. For all IFA scale bar (white line) denotes 10 μm. Significance was determined by Mann-Whitney t-test, ** denotes $p<0.01$.

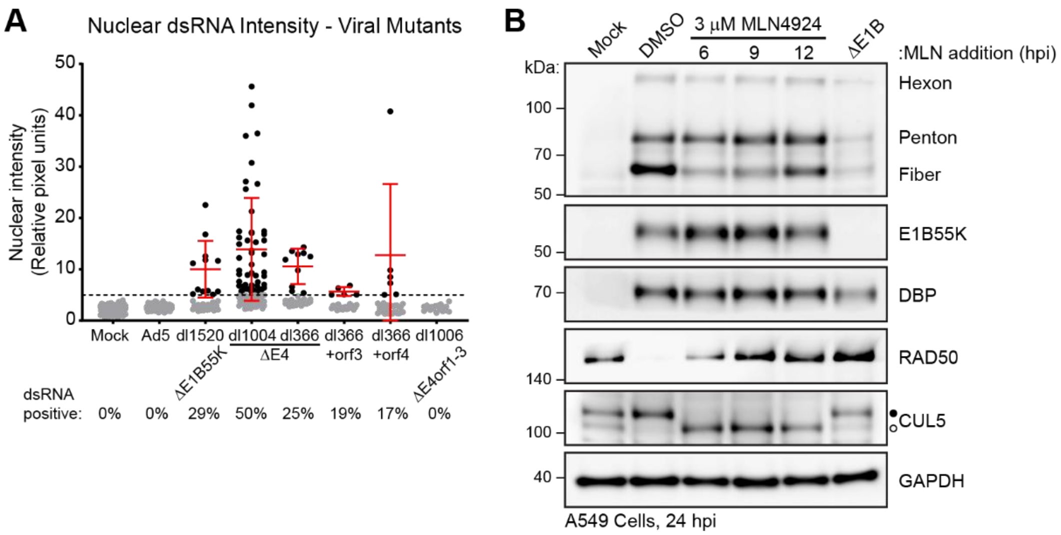

Supplementary Figure 2. dsRNA is only produced when viral ubiquitin ligase is absent. (A) A549 cells were infected with the designated Ad5 mutant viruses for 48 hours and analyzed by IFA for nuclear dsRNA using J2 antibody. Nuclear dsRNA was quantified as MNFI and every cell displayed as a dot. The dsRNA positive threshold was defined as 4 standard deviations over the MNFI of dsRNA in uninfected cells and displayed as a dashed line. Cells below the threshold were colored grey, while cells positive for nuclear dsRNA were colored black and the mean and standard deviation of these positive cells is denoted by red error bars. The percentage of total cells expressing dsRNA during infection with each virus is shown below their name on the x-axis. **(B)** Immunoblot analysis following infection of A549 cells with wildtype Ad5 (WT Ad5) or ΔE1B55K (ΔE1B) mutant virus. WT infected cells were treated with DMSO or 3 μM NEDDylation inhibitor MLN4924 at the times indicated post infection and all lysates were harvested 24 hpi. ● indicates low mobility NEDDylated CUL5, while ○ indicates the unmodified form.

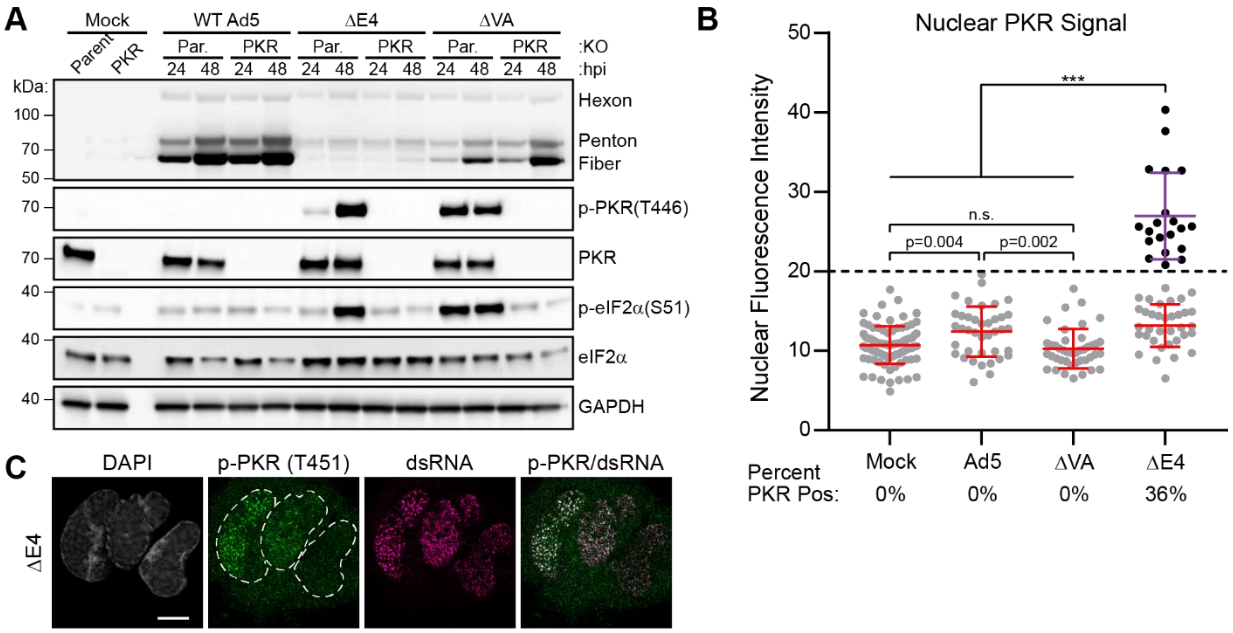

Supplementary Figure 3. Effects of activated PKR in the nucleus during ΔE4 infection. (A) A549 cells (Parent, Par.) were transduced with PKR-targeting gRNA to make a CRISPR/Cas9-mediated PKR knockout (KO) cell line. This clone results in total loss of PKR protein and loss of eIF2α activation downstream of AdV infection. While PKR KO partially rescues the late protein (Hexon, Penton, Fiber) defect of ΔVA virus, it was not sufficient to rescue ΔE4 virus infection. **(B)** Nuclear total PKR from **Figure 5A** was quantified as MNFI and every cell displayed as a dot. The positive threshold was defined as 4 standard deviations over the MNFI of nuclear PKR in uninfected cells and displayed as a dashed line. Cells below the threshold were colored grey, while cells positive for nuclear PKR were colored black. The mean and standard deviation of negative cells (background fluorescence) is denoted by red error bars, while the mean and standard deviation of cells enriched for nuclear PKR is denoted by purple error bars. The percentage of total cells expressing nuclear PKR during infection with each virus is shown below their name on the x-axis. **(C)** A549 cells were infected with ΔE4 virus for 38 hours and stained for activated PKR (green) and 9D5 anti-dsRNA antibody (magenta). Nuclei were stained with DAPI, and nuclear periphery outlined by a dashed white line. Scale bar (white line) displays 10 μm.

	Name	Forward Primer (5' to 3')	Reverse Primer (5' to 3')
qRT Primers	TPL Spliced	TTCCGCATCGCTGTCTG	CCGATCCAAGAGTACTGGAAAG
	TPL Unspliced	GTCCAGGGTTTCCTTGATGAT	
	Fiber Spliced	GAAAGGCGTCTAACCAGTCA	AAAGGCACAGTTGGAGGAC
	Fiber Unspliced	CATCCGCACCCACTATCTTC	
	E1A Spliced	TGGACCCTCGGGAATGAA	TCAGGCTCAGGTTCAGACA
	E1A Unspliced	GGCTTAAGGGTGGGAAAGAA	
	VA RNA	ACTCTTCCGTGGTCTGGTGGATAA	TTGTCTGACGTCGCACACCT
	GAPDH	TGCACCACCAACTGCTTAGC	GGCATGGACTGTGGTCATGAG
	HPRT1	TGACACTGGCAAAACAATGCA	GGTCCTTTTCACCAGCAAGCT
	DESI1	GTGTGCAGTGGCATGATCTC	ACATGGCAAAACCCTGTCTC
	PEX26	CTCCCGAGTAGCTGGGACTA	CGATGGCTCACACCTGTAAT
	mt-CO1	ACGTTGTAGCCCACTTCCAC	TGGCGTAGGTTTGGTCTAGG
	mt-ND5	TCGAAACCGCAAACATATCA	CAGGCGTTTAATGGGGTTTA
Morpholinos	GFP NTC	ACAGCTCCTCGCCCTTGCTCACCAT	
	TPL Exon 2 Donor	TCTACATGCTAGGCTCTTACCGTTC	
	TPL Exon 3 Donor	CACGGTGCTCAGCCTACCTTG	
	Fiber Acceptor	ATCTGCAACAACATGAAGATAGTGG	

Supplementary Table 1. Oligonucleotides used in this study. List of DNA oligonucleotides used for qRT-PCR and phosphorodiamidate morpholino oligomers (Morpholinos) used to block splicing reactions.
